# Role of Electrolyte pH on Water Oxidation for Iridium Oxides

**DOI:** 10.1021/jacs.3c12011

**Published:** 2024-03-25

**Authors:** Caiwu Liang, Yu Katayama, Yemin Tao, Asuka Morinaga, Benjamin Moss, Verónica Celorrio, Mary Ryan, Ifan E. L. Stephens, James R. Durrant, Reshma R. Rao

**Affiliations:** † Department of Materials, Imperial College London, Exhibition Road, SW72AZ London, United Kingdom; ‡ Department of Chemistry, Centre for Processable Electronics, Imperial College London, White city campus, W12 0BZ London, United Kingdom; § Department of Energy and Environmental Materials, SANKEN (The Institute of Scientific and Industrial Research), Osaka University, Mihogaoka 8-1, Osaka 567-0047, Ibaraki, Japan; ∥ Diamond Light Source, Harwell Science and Innovation Campus, Didcot OX11 0DE, United Kingdom

## Abstract

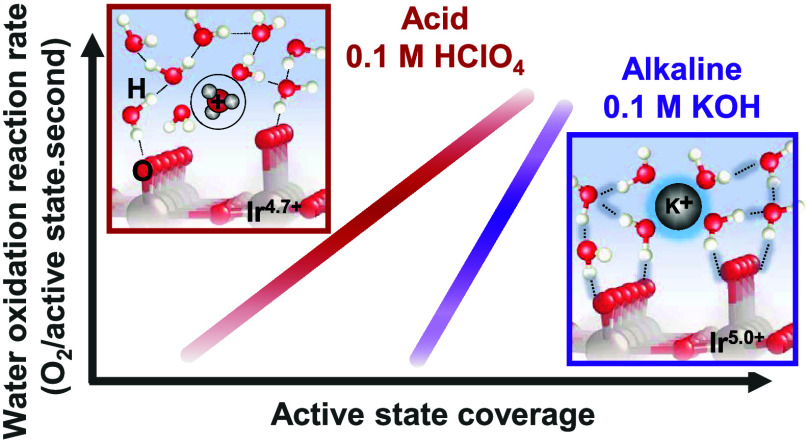

Understanding the effect of noncovalent interactions of intermediates at the polarized catalyst–electrolyte interface on water oxidation kinetics is key for designing more active and stable electrocatalysts. Here, we combine *operando* optical spectroscopy, X-ray absorption spectroscopy (XAS), and surface-enhanced infrared absorption spectroscopy (SEIRAS) to probe the effect of noncovalent interactions on the oxygen evolution reaction (OER) activity of IrO_*x*_ in acidic and alkaline electrolytes. Our results suggest that the active species for the OER (Ir^4.x+^–*O) binds much stronger in alkaline compared with acid at low coverage, while the repulsive interactions between these species are higher in alkaline electrolytes. These differences are attributed to the larger fraction of water within the cation hydration shell at the interface in alkaline electrolytes compared to acidic electrolytes, which can stabilize oxygenated intermediates and facilitate long-range interactions between them. Quantitative analysis of the state energetics shows that although the *O intermediates bind more strongly than optimal in alkaline electrolytes, the larger repulsive interaction between them results in a significant weakening of *O binding with increasing coverage, leading to similar energetics of active states in acid and alkaline at OER-relevant potentials. By directly probing the electrochemical interface with complementary spectroscopic techniques, our work goes beyond conventional computational descriptors of the OER activity to explain the experimentally observed OER kinetics of IrO_*x*_ in acidic and alkaline electrolytes.

## 1 Introduction

Oxygen evolution reaction (OER) is the key anodic reaction for several energy conversion and storage technologies, including but not limited to CO_2_ reduction to liquid fuels and green hydrogen generation via water electrolysis. Despite its significance, the kinetics of this reaction are sluggish, primarily attributed to its multistep proton-coupled electron transfer mechanism and the presence of various intermediate states during the reaction.^1,2^ Seminal theoretical work by Rossmeisl and Nørskov has shown that the energetics of intermediates that form during the water oxidation reaction, particularly *OH, *O, and *OOH, can dictate the catalytic activity.^3,4^ These intermediates form at complex, polarized interfaces, and the search for new catalysts has largely focused on optimizing the binding energetics of these states by changing material chemistry.^5,6^ In contrast, the role of the interfacial solvent environment at such polarized interfaces in controlling these energetics, and thus the OER reaction kinetics, remains poorly understood and is the focus of this study.

Recent research has shown that modeling the interaction between the catalyst surface and isolated reaction intermediates cannot sufficiently describe the difference in OER reaction kinetics as a function of electrolytes for a number of oxides and oxyhydroxides.^7−18^ Noncovalent interactions between intermediates, ions in the electrolyte, and interfacial water molecules can play a crucial role in altering the (1) density of accessible surface sites,^15,19^ (2) energetics of intermediates,^20,21^ and/or (3) kinetics of the reaction, thus changing the rate of the water oxidation reaction.^22−24^ However, gaining atomic level insights into these interfaces is challenging considering that the hydrogen-bonding environment of water molecules at electrified interfaces is very different from that of bulk water.^19,25,26^ For example, at Pt/water and Au/water interfaces, the orientation and degree of ordering of interfacial water molecules is significantly different as a function of pH and potential.^23,27−33^ This interfacial water layer structure is also affected by the cations and anions in the electrolyte due to the difference in strength of electrostatic interaction or hydration ability of the ions with water.^31,34^ For OER occurring on metal oxides, the surface polarity,^15,35,36^ and the formation of intermediates on the surface as a function of potential^37,38^ can also influence the interfacial solvent environment. However, molecular understanding of the role of noncovalent interactions at oxide/water interfaces and their impact on the energetics of different intermediates and the OER reaction kinetics is currently still lacking.

IrO_*x*_ exhibits benchmark OER activity in acid and has served as a model catalyst for understanding the water oxidation mechanism.^39^ Recent studies have demonstrated the critical, but unexplained, role of the electrolyte in determining the redox potentials and OER reaction kinetics for iridium-based oxides at different pH. For example, work by Suntivich and co-workers^9^ on single-crystal IrO_2_(110) has shown that the redox peaks in cyclic voltammetry shift to lower potential with increasing pH, from ∼1.20 V_RHE_ and ∼1.63 V_RHE_ in acid (0.1 M HClO_4_) to ∼0.90 V_RHE_ and ∼1.53 V_RHE_, respectively, in alkaline (0.1 M KOH). They attributed this cathodic shift in redox peak potential to stronger binding of oxygenated intermediates, *OH and *O, at pH 12.9 compared to that at pH 1. However, the authors found that the overpotential difference of only ∼30 mV at a current density of 5 μA cm_geo_^–2^ in acidic and alkaline electrolytes cannot be explained by the energetic differences of *OH and *O determined by the redox peak positions, suggesting the importance of additional electrolyte effects. Similarly, on electrodeposited IrO_*x*_, the redox peaks were found to shift negatively with approximately 30 mV/pH from pH 2 to 12, while the overpotential at 10 μA cm_geo_^–2^ for all of the pHs was relatively similar.^40^ Recently, Nong et al.^41^ have demonstrated, from DFT calculations, that the binding energy of *O can be weakened with increasing coverage of these species due to the repulsive adsorbate–adsorbate interactions, resulting in an increase in the kinetics of the rate-determining O–O bond formation step with increasing coverage of *O. In our recent work, we quantified the interaction strength between *O species using *operando* optical spectroscopy and revealed how this interaction strength controls the intrinsic reaction kinetics of water oxidation.^42−44^ Nong et al. suggested that this adsorbate–adsorbate interaction between *O species, which are crucial for OER kinetics, is mediated via the electrolyte.^41^ Therefore, based on previous observations of electrolyte-dependent OER kinetics on IrO_2_, we can hypothesize that the electrolyte is crucial in not only changing binding energetics at low coverage^9,40^ but also altering intersite interactions.^41^ However, the molecular details of how the interfacial electrolyte modulates the binding energetics and/or the interaction between adsorbates are not known, which limits the development of more active electrochemical interfaces.

Here, we experimentally identify and quantify the density of potential-dependent intermediates, the interactions between them, and the reaction kinetics of IrO_*x*_ under acid and alkaline conditions using *operando* optical spectroscopy and X-ray absorption spectroscopy (XAS). We also probe the interfacial water structure using *operando* surface-enhanced infrared absorption spectroscopy (SEIRAS). Our results suggest that the active states for the OER (Ir^4.x+^–*O) form at a much less positive potential in alkaline (∼1.1 V_RHE_) compared to acid (∼1.32 V_RHE_), while the interaction parameter between these species is ∼0.2 eV higher in alkaline electrolytes. We attribute these differences to the larger fraction of water within the hydration shell of cations at the interface in the alkaline 0.1 M KOH electrolyte, compared to the acidic 0.1 M HClO_4_ electrolyte, which can stabilize oxygenated intermediates and facilitate long-range interactions between them. By directly probing the electrified solid–liquid interface, we rationalize the trends observed for redox peak potentials and the OER activity in acid and base for IrO_*x*_. Our work unravels a new fundamental understanding of the role of interfacial electrolytes in controlling OER kinetics and thus provides insights into how the solvent effects can be exploited to increase catalytic activity.

## 2 Results and Discussion

### 2.1 pH-Dependent Redox and OER Activity

**Figure 1:**
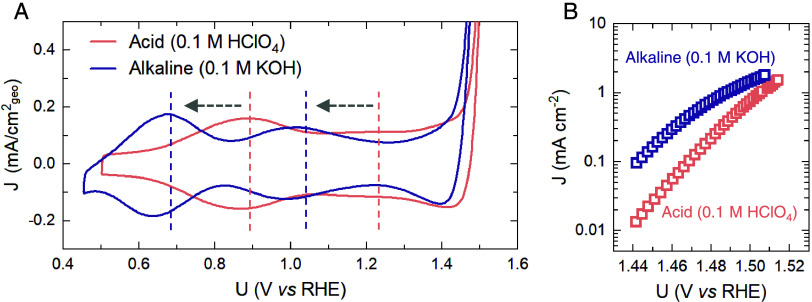
Electrochemistry of IrO*_x_* under acid and alkaline conditions. (A) Comparison of cyclic voltammograms of IrO_*x*_ in 0.1 M HClO_4_ (pink) and 0.1 M KOH (purple) at a scan rate of 10 mV s^–1^ at room temperature. Iridium oxide samples were deposited on a ∼1 cm × 1 cm area of FTO substrates. The measurement was conducted in a typical three-electrode setup using an SP-150 Biologic potentiostat, with Pt mesh and a homemade reversible hydrogen electrode (RHE) as counter and reference electrodes, respectively (see Electrochemical Measurement section in the Supporting Information). The dashed lines indicate the redox peak positions. (B) Tafel plot obtained from CV data on the left; the current is obtained by an average of forward and backward scans to minimize the effect of redox capacitance background.

Hydrous, amorphous iridium oxide films were prepared by electrodeposition on FTO substrates, using the same method described in our previous work^42^ (see Methods section in the SI). The IrO_*x*_ films consist of 100–200 nm diameter nanoparticles and are XRD amorphous.^42^ Cyclic voltammograms measured on these films (Figure 1A) show peaks at ∼0.9 V_RHE_ and ∼1.22 V_RHE_ in acidic conditions (0.1 M HClO_4_, pH ∼ 1.0) and at ∼0.68 V_RHE_ and ∼1.03 V_RHE_ in alkaline conditions (0.1 M KOH, pH ∼ 12.9). These two peaks are attributed to deprotonation of *H_2_O at a coordinatively unsaturated (CUS) Ir site (H_2_O_(cus)_ →*OH_(cus)_+ H^+^ + e^–^) and deprotonation of the bridging oxygen (*OH_(cus)_ + *H_b_ → *OH_(cus)_ + H^+^ + e^–^ + *), respectively, based on our previous work.^42^ The cathodic shift of redox peak positions in alkaline electrolytes is in agreement with reports on IrO_2_(110)^9,40^ and electrodeposited IrO_*x*_.^40^ Our previous work^42^ in 0.1 M HClO_4_ has shown using optical spectroscopy that a third redox transition occurs at ∼1.4 V_RHE_, attributed to the formation of *O on the Ir CUS site (*OH_(cus)_ →*O_(cus)_ + H^+^+ e^–^). However, based on electrochemical techniques alone, its peak position cannot be extracted due to the overlap of the OER catalytic current and redox capacitance current. We find that the positions of these redox peaks are mainly dependent on electrolyte pH, with negligible effect on the cation used in the electrolyte (Figure S1). Despite the substantial difference in redox transition peaks (>200 mV), the difference in OER activity, normalized by geometric current density, is much smaller, with the overpotential at 0.5 mA cm_geo_^–2^ in alkaline (∼1.46 V_RHE_) being only 30 mV lower than that in acid (∼1.49 V_RHE_; Figure 1B). This observation is also in agreement with previous reports on iridium oxides.^9,40^ Thus, both our observation and previous reports show a similar overpotential but a shift in redox peak to stronger binding in 0.1 M KOH, which is inconsistent with a simple density functional theory (DFT)-derived volcano model based on the single-site mechanism, neglecting adsorbate–adsorbate interactions.^3,4,45^ Therefore, in order to further investigate this discrepancy and unravel the role of the electrolyte in governing the reaction energetics and kinetics, we use a combination of optical, X-ray, and vibrational spectroscopy.

### 2.2 Quantifying the Density and Energetics of Redox Active Species

**Figure 2:**
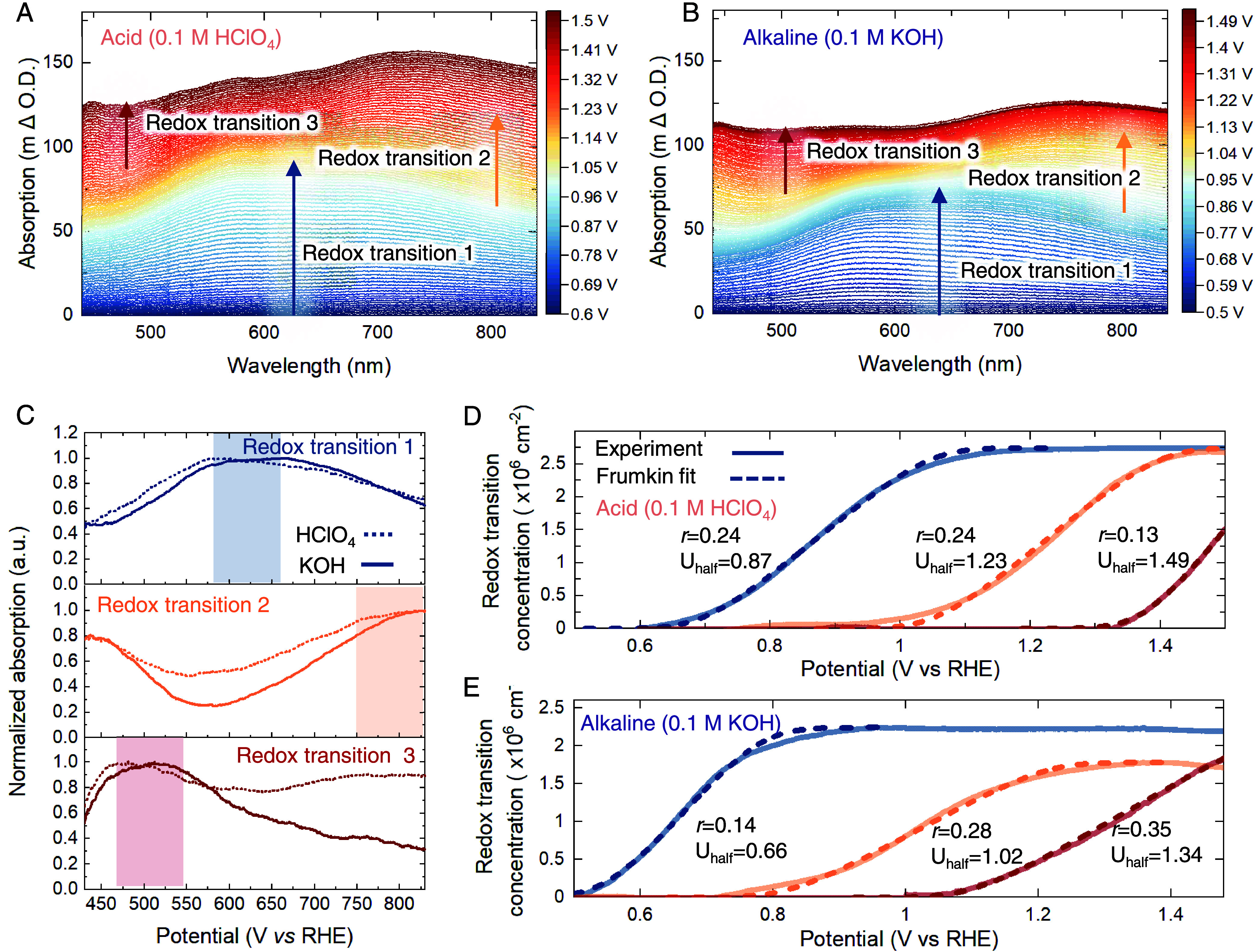
Redox features and concentrations as a function of potential for IrO*_x_* at acid and base conditions. (A) Differential absorption spectra of IrO_*x*_ during a linear sweep scan from 0.6 V_RHE_ to 1.53 V_RHE_ in 0.1 M HClO_4_ at a scan rate of 1 mV s^–1^ at room temperature (*iR* corrected). Absorption changes were recorded at every 1 mV and shown every 10 mV (see Figure S3 for full spectra). The absorption changes are calculated with respect to the absorption at 0.60 V_RHE_. (B) Differential absorption spectra of IrO_*x*_ during a linear sweep scan from 0.50 V_RHE_ to 1.53 V_RHE_ in 0.1 M KOH. (C) Comparison of differential absorption spectra for each redox transition in 0.1 M HClO_4_ and 0.1 M KOH. Concentration of redox transitions that have completed as a function of potential (solid line) and the corresponding Frumkin isotherm fitting for IrO_*x*_ in (D) 0.1 M HClO_4_ and (E) 0.1 M KOH electrolytes.

To quantify the potential-dependent density of active states and their intrinsic rate of reaction, we used time-resolved operando optical spectroscopy. Figure 2A shows the change in optical absorption spectra (vs 0.6 V_RHE_) when the potential is increased in a 10 mV step, indicating alterations in the surface speciation of the iridium oxides in response to the varying potential. On increasing the potential in the acidic electrolyte, broad absorption bands at ∼650, ∼800, and ∼500 nm are observed at potential ranges of 0.6 V_RHE_–1.0 V_RHE_, 1.0 V_RHE_–1.3 V_RHE_, and >1.3 V_RHE_, respectively, in agreement with our previous reports.^42,43^ Optical spectroscopy measurements in an alkaline electrolyte show three similar absorption bands with increasing potential. However, the potential range for the formation of these spectral features are lower, 0.5 V_RHE_–0.8 V_RHE_, 0.8 V_RHE_–1.15 V_RHE_, and >1.15 V_RHE_, respectively (Figure 2B). We confirm that these variations mainly arise from the shift in pH as adding cations (K^+^) into the electrolyte shows a negligible effect on the spectra (Figure S2). Similar results have been observed previously on RuO_2_(110) single-crystal surfaces, where it was found that introducing various cations (Li^+^, K^+^, and Na^+^) into acidic electrolytes did not change the electrochemistry, while these cations show stronger influence on electrochemistry when added to alkaline electrolytes.^12^ To analyze the concentration of each redox state as a function of potential, we deconvoluted the total absorption using a linear combination fitting method, reported in our previous work (see Supporting note 1 for deconvolution details).^42^ The three distinct spectral shapes used for the deconvolution can be obtained for both acid and alkaline conditions by using differential analysis. We note that the spectral shapes of the three states in acid and base are similar, with absorption bands in the alkaline electrolyte slightly shifted to higher wavelengths, indicating similar intermediate states in acid and alkaline (Figure 2C, see Figure S4 for details of differential analysis). The resultant deconvoluted absorption can be converted to a concentration of redox states using the Beer–Lambert law and the measured correlation between the change in absorption and charge passed in a pulsed voltammetry measurement in a potential regime, where only one redox transition occurs (see Figures S5–S7 and Table S1 for details). With the above deconvolution, the concentration of each redox transition as a function of potential can be obtained (Figure 2D,E). The saturated concentrations of the redox active states are similar in acid and alkaline electrolytes, suggesting that the density of accessible redox centers in IrO_*x*_ is relatively independent of pH. In addition, a simple calculation comparing the theoretical Ir site concentration in the film with the measured saturated concentration (∼2.75 × 10^16^ cm^–2^) suggests that most of Ir sites in the porous amorphous IrO_*x*_ are redox-active (see Supporting Note 2 and Figure S8 for details). The redox peak positions for the first and second redox transitions are 0.87 V_RHE_ and 1.23 V_RHE_ in acid and 0.66 V_RHE_ and 1.02 V_RHE_ in alkaline electrolytes. The optically measured redox peak positions match the values observed in the cyclic voltammogram: ∼0.9 V_RHE_ and ∼1.22 V_RHE_ in acid and ∼0.68 V_RHE_ and ∼1.03 V_RHE_ in alkaline electrolytes. In addition, using optical spectroscopy, we can determine a third redox transition at a high potential, with the redox peak at ∼1.49 V_RHE_ in acid and ∼1.34 V_RHE_ in alkaline electrolytes. A detailed comparison of the redox waves obtained from optical and electrochemical signals is shown in Figure S9.

**Figure 3:**
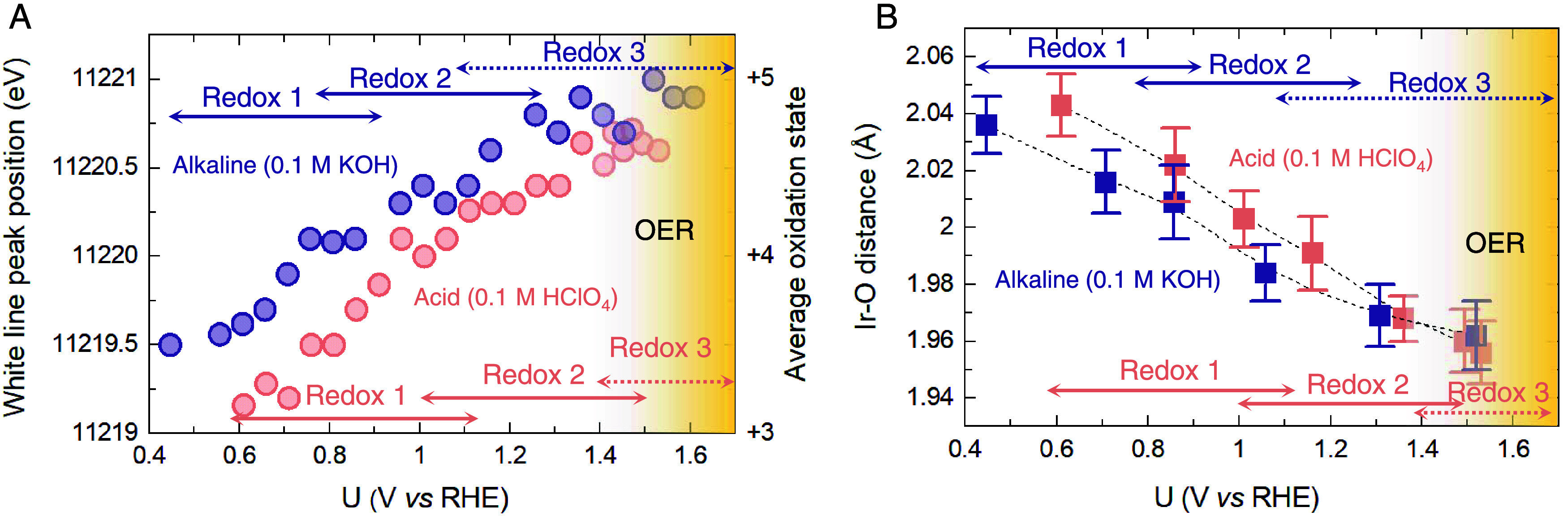
Electronic structure and coordination environment of iridium centers. (A) White line position and its corresponding average Ir chemical state of IrO_*x*_ at different potentials in 0.1 M HClO_4_ (red circle) and 0.1 M KOH (purple circle). The measurements were performed using a home-built operando XAS cell that enables us to measure the electrodeposited film on FTO, the same type of sample as used in the operando optical measurement (Figure S10). The average chemical states were calculated based on the while line position shift and an increase of around 1.0 eV per d-band hole calibrated from reference metallic iridium Ir^0^(5d^7^), IrCl_3_ (5d^6^), and IrO_2_(5d^5^) (see Figures S11 and S12 for details). (B) Ir–O bond distances as a function of potential in acid and alkaline, obtained by the fitted *k*^2^-weighted Fourier transforms of EXAFS. The arrows in both figures indicate the potential window of redox transitions 1, 2, and 3 observed in optical spectroscopy (Figures S14 and S15, and Table S2). Redox 1, 2, and 3 represent the first, second, and third redox transition.

The onset of redox processes at less positive potentials in the alkaline KOH electrolyte compared to the acidic HClO_4_ electrolyte was further confirmed using *in situ* X-ray absorption spectroscopy (XAS). XAS measurements were performed on electrodeposied IrO_*x*_ film on FTO to be consistent with the optical spectroscopy and electrochemical measurements, (see Figure S10 for cell design). The white line position of the Ir-L_3_ edge in the X-ray absorption near-edge spectroscopy (XANES) spectra (Figure S11) shifts to higher energy with increasing potential in acid and alkaline electrolytes. As shown in Figure 3A, with the potential increasing from 0.5 V_RHE_ to 1.6 V_RHE_, the average Ir oxidation state increases from 3.5 to 5.0 in the alkaline electrolyte and 3.1 to 4.7 in the acidic electrolyte based on calibration using iridium standards (Figure S12). The increase of the oxidation state with the applied potential is also confirmed by the increasing integral area of Ir-L_3_ white line with the potential (Figure S13). We note that the oxidation state observed here is an average value across the sample and the oxidation state at the surface/active areas may be higher. Combining the *operando* XANES with our previous DFT calculations,^42^ the three redox transitions can be assigned to successive deprotonation steps that are accompanied by an increase in oxidation state of iridium or surface oxo groups (redox transition 1: *H_2_O _(cus)_ → *OH_(cus)_ + H^+^ + e^–^ (acidic), *H_2_O_(cus)_ + OH^–^→ *OH_(cus)_ + H_2_O + e^–^ (alkaline); redox transition 2: *OH_(cus)_ + *H_b_ → *OH_(cus)_ + H^+^ + e^–^ + * (acidic), *OH_(cus)_ + *H_b_ + OH^–^→ *OH_(cus)_ + H_2_O + e^–^ + * (alkaline); and redox transition 3: *OH_(cus)_ →*O_(cus)_ + H^+^ + e^–^ (acidic), *OH_(cus)_ + OH^–^ → *O_(cus)_ + H_2_O + e^–^ (alkaline)). It is evident that at a given potential, IrO_*x*_ is more oxidized (i.e., iridium has a higher oxidation state) in alkaline electrolytes compared to acid, in agreement with our electrochemistry and optical spectroscopy results. At potentials >1.4 V_RHE_, the Ir oxidation state begins to plateau, with the oxidation state being higher in alkaline electrolytes compared to acid. Prior work has suggested the presence of higher oxidation states of iridium at this potential region;^46,47^ however, recent reports also suggest that the redox transitions at a high potential correspond, in part, to the formation of holes on surface oxo groups.^35,48,49^ The trends observed in the XANES spectra are also consistent with the shortening of the Ir–O bond with increasing potential, as observed in the X-ray absorption fine structure (EXAFS). The Fourier transforms of EXAFS spectra and the simulations of the spectra (Figures S14–S15 and Table S2) revealed the presence of characteristic Ir–O bond distances and very weak Ir–Ir interaction peaks, showing a short-range disorder structure of amorphous IrO_*x*_. Figure 3B shows that the Ir–O bond significantly shortens from ∼2.04 to ∼1.96 Å with increasing potential in both acid and alkaline electrolytes. However, at a given potential, the Ir–O bond is shorter in alkaline electrolytes compared to acid electrolytes, with the Ir–O bond distance becoming similar at the potential >1.3 V_RHE_. The shortening of the metal–oxygen bond distance with increasing redox state of the central metal ion due to a decrease in effective ionic radius has been widely reported.^50,51^ Therefore, the XANES and EXAFS measurements further validate that the potential at which iridium centers are oxidized is lower in alkaline electrolytes compared to acid.

In addition to the oxidized states forming at a lower potential in alkaline electrolytes, the concentration of redox states as a function of potential shows a significantly different slope compared to that in acidic electrolytes (Figure 2D,E). Assuming the saturated concentration of redox states observed in our optical signal is the full coverage of the electrochemically active states on IrO_*x*_, the coverage θ of each redox state at a given potential can be calculated as a fraction of full coverage (i.e., θ = *D*/*D*_max_, where *D* is the density of states and *D*_max_ is the maximum saturating density). Thus, we can analyze the electroadsorption isotherm of each redox transition using *U* vs θ data. We find that the *U* vs θ data cannot be fitted using a simple Langmuir isotherm (Figures S16 and S17), which assumes no interaction between adsorbates, as also demonstrated in our previous work in acidic electrolytes.^42^ Instead, the electroadsorption isotherms can be modeled using a Frumkin isotherm, with *R*^2^ as high as ∼0.99 (Figures 2D,E, S16 and S17). We note that a similar trend of Frumkin interaction parameters was also observed on rutile iridium oxide in our previous work,^42^ indicating that although IrO_*x*_ is a highly porous catalyst, its redox transition processes are similar to the dense rutile iridium oxide film and is thus most likely related to the adsorption process of oxygenated species, as opposed to a slower, battery-like intercalation process, where the reaction is limited by solid-state diffusion.^52^ The Frumkin isotherm suggests the existence of lateral interactions of the adsorbed intermediates that lead to coverage-dependent binding energetics for the adsorbates, i.e., Δ*G*_state(θ)_^0^ = Δ*G*_state(θ=0)_^0^ + *r*θ, where *r* (in eV) is the interaction energy of the absorbates at full coverage, θ is the coverage of the absorbates (0 < θ < 1), and Δ*G* is the Gibbs free energy for adsorption. The parameter *r* was obtained by fitting the *U* vs θ data and has been labeled in Figure 2D,E (see Supporting note 3 for details). Recent work by Nong et al.^41^ on IrO_*x*_ has proposed that this interaction between adsorbates on the surface is mediated through the electrolyte. We have shown that the interaction parameter was independent of the degree of crystallinity of iridium oxide.^42^ However, here, we see that the interaction energy *r* differs significantly for the redox transitions in acidic and alkaline electrolytes. The interaction energy for the first redox transition is 0.24 and 0.12 eV for acidic and alkaline electrolytes, respectively, while these values are more comparable for the second redox transition at 0.24 and 0.28 for acid and alkaline electrolytes, respectively. Finally, at OER-relevant potentials, the interaction energy of the third redox reaction, i.e., between adsorbed Ir^4.x+^ (*O) species, in acidic electrolytes is 0.13 eV, much smaller than that observed in alkaline electrolytes (0.35 eV). This indicates much stronger, repulsive interactions between neighboring catalytically active state Ir^4.x+^ (*O) (state generated from redox transition 3) in the alkaline electrolyte compared to the acidic electrolyte. Therefore, using a combination of time-resolved optical spectroscopy and X-ray absorption spectroscopy, we demonstrate pH-dependent interaction energy between adsorbates, which can explain the difference in redox transitions and OER activity in acid and alkaline electrolytes.

### 2.3 Unraveling the Interfacial Water Structure in Acidic and Alkaline Electrolytes

**Figure 4:**
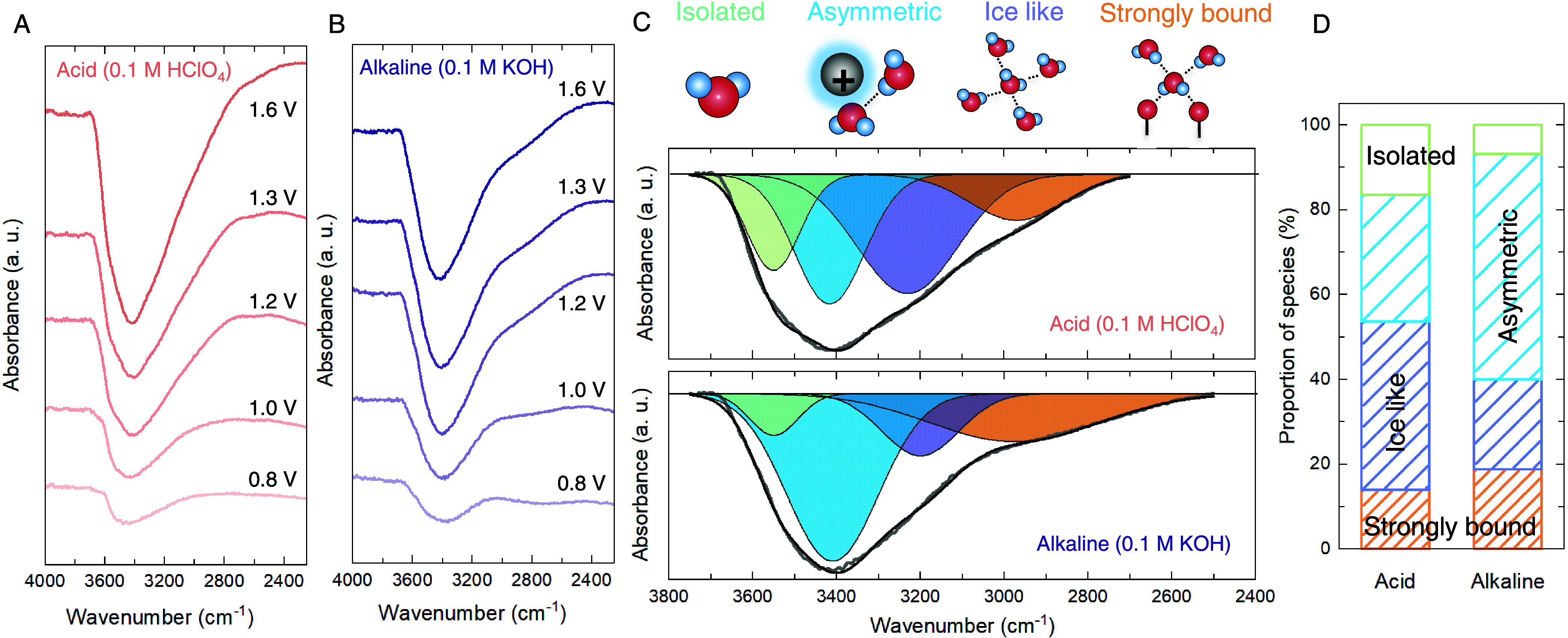
Water layer structure at the interface of IrO*_x_*. (A) ATR-SEIRAS spectra of the potential-dependent behavior of interfacial water in 0.1 M HClO_4_ solution in the O–H stretching regime. Reference spectrum is taken at 0.6 V_RHE_. (B) ATR-SEIRAS spectra of the potential-dependent behavior of interfacial water in a 0.1 M KOH solution. The reference potential is 0.6 V_RHE_. The baseline of each spectrum was corrected using OMNIC software with a three-point autocorrection method. (C) Deconvolution of the O–H stretching vibration peak at 1.6 V_RHE_ in 0.1 M HClO_4_ (top) and 0.1 M KOH (bottom) solutions. (D) Quantification of the fraction of the different water species at 1.6 V_RHE_. Quantified results for other potentials are exhibited in Figure S22.

The results above show that the binding energetics of the surface adsorbates and the interaction strength between them, on IrO_*x*_, are significantly different in acid and base, which suggests that these two parameters are controlled by the IrO_*x*_–electrolyte interface, where noncovalent interactions between the electrolyte and intermediates can play a key role. The pH of the electrolyte has been reported to significantly affect the electrochemical double layer (EDL) structure and the intermediate adsorption energy.^9,19,53,54^ To understand how the electrolyte affects the energetics of intermediates on IrO_*x*_ in acid and alkaline, we probed the interfacial water structure using in situ surface-enhanced infrared absorption spectroscopy (SEIRAS) in ATR mode, where amorphous IrO_*x*_ was deposited on a Pt conductive layer. The deposited film shows characteristic redox peaks from amorphous IrO_*x*_ in CV, in contrast to that of the Pt surface, indicating a minimal effect of the Pt underlayer on the measurements (Figure S18). The ATR-SEIRAS results enhance the signal within ∼5–10 nm from the Pt surface, thus enabling the detection of water structure at the interface of IrO_*x*_ located at this regime. Figure 4A,B shows the in situ SEIRAS spectra of interfacial water molecules on IrO_*x*_ at various potentials in 0.1 M HClO_4_ and 0.1 M KOH solutions, respectively, for the O–H stretching vibration mode (2500–4000 cm^–1^) (see Figure S19 for the full spectra at every 50 mV). The spectra of the Pt substrate were also recorded, which are significantly different from those of IrO_*x*_ (Figures S20 and S21A), suggesting that the spectral features are mainly from the IrO_*x*_/electrolyte interface. The frequency of the O–H stretching is closely related to the covalency, or the distance between O and H atoms, in the water molecules; a higher covalency of the O–H bond results in a lower vibration distance and thus a higher stretching frequency. Previous studies have shown that an increase in the hydrogen bonding (H–O–H···OH) between water molecules results in an increase of the O–H bond distance and thus a decrease in the O–H stretching frequency.^54−56^

The O–H stretching region between 2500–3900 cm^–1^ can be deconvoluted into four different types of water molecules at the interface.^27,57−59^ As shown in Figure 4C, the peak centered at the highest wavenumbers ∼3610 cm^–1^ is assigned to isolated water molecules that do not interact strongly with neighboring water molecules (zero hydrogen bonds).^27,28,53^ The peak at ∼3400 cm^–1^ is assigned to asymmetric H-bonded water molecules within the hydration shells of cations that cannot form a complete H-bonding network (one to three hydrogen bonds) with neighboring water molecules due to interaction with cations.^53,12,60^ The peak at ∼3200 cm^–1^ is assigned to symmetric H-bonded water molecules (ice-like) at the interface, where the hydrogen-bonding structure is similar to that of bulk water molecules, with four hydrogen bonds formed per water molecule.^12,59^ Finally, the peak at the lowest wavenumber ∼2900 cm^–1^, which is usually absent for crystalline metals and metal oxides,^12,56,61^ is assigned as strongly bound water within the hydrated structure of IrO_*x*_. This distinct signal of strongly bound water was also observed during the electrodeposition phase of IrO_*x*_, where the signal at ∼2900 increases rapidly during the initial stages of electrodeposition (see Figure S21B). This distinct signal, observed on IrO_*x*_ before the OER measurement conducted in acidic or alkaline electrolyte, supports our assignment of strongly bonded water within the structure. A similar peak was reported on as-prepared hydrated iron oxides, which was absent after annealing to higher temperatures.^62^

Figure 4D shows that asymmetric water molecules dominate the interfacial water structure under alkaline conditions. This is consistent with previous work that has estimated the cation concentration at polarized interfaces to be ∼80 times that of the bulk.^32^ On the other hand, the interfacial water structure in acidic electrolytes is composed primarily of ice-like and isolated water molecules. These differences in the nature and ordering of interfacial ions and water molecules might be related to the change in the point of zero charge as a function of pH, as has been demonstrated for metal surfaces.^23,30,53,60^ However, a direct measure of the point of zero charge of complex oxide surfaces and its relation to the interfacial water structure and reaction kinetics requires further investigation. The ordered interfacial structure in the alkaline electrolyte consists of alkali metal cations, which have a stronger hydration ability leading to stronger polarized water and a larger hydration shell with multiple water molecules, compared to hydronium ions present in the acidic electrolyte.^63^ Thus, these metal cations, with stronger polarized water molecules, enable the formation of a stronger absorbate–water–cation interaction network in alkaline compared with the acidic electrolyte. This is also consistent with the cation dependence of OER kinetics in the alkaline electrolyte that has been observed on IrO_2_(110),^9^ RuO_2_(110),^12^ and NiOOH,^14^ where it has been hypothesized that the nature of the interfacial cations and the water molecules within their hydration shell plays an important role in dictating the overall OER activity.

**Scheme 1:**
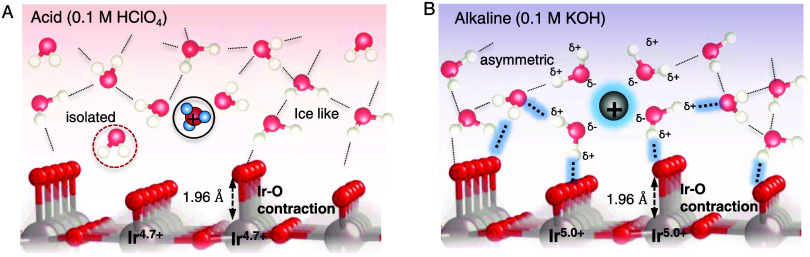
Schematic of Electrochemical Interface under Acidic 0.1 M HClO_4_ Electrolyte (A) and Alkaline 0.1 M KOH Electrolyte (B) during Water Oxidation at ∼1.5 V_RHE_ Dashed line indicates the hydrogen bond between O and H at the polarized interface. We note that amorphous IrO*_x_* is expected to have short-range order. However, for simplicity of illustrating the interface, the surface has been depicted to be that of IrO_2_(110). A more clear schematic of the bulk structure of amorphous IrO*_x_* highlighting these short-range order features in our previous work^42^ and is shown in Figure S23.

We can attribute the differences in binding energetics and interaction parameters in acidic and alkaline electrolytes observed from our optical and electrochemical data to these differences in the interfacial water structure determined from our SEIRAS data. In the alkaline electrolyte, the stronger interactions between the water molecules within the hydration shells of cations and surface intermediates offer greater stabilization of intermediates and thus make it easier to form the oxidized species (less positive potential of redox transitions in alkaline compared to acidic electrolyte). Considering the positive charge of K^+^, the water within its hydration shell can interact strongly with it, as demonstrated by scattering, spectroscopy, and simulations.^64^ Consequently, water within the hydration shell of K^+^ is polar, with the oxygen atoms having a net negative and hydrogen atoms having a net positive charge due to their strong interaction with the cation.^63−65^ The higher fraction of these polar water molecules at the interface provides a more rigid interfacial hydrogen-bonding network (Scheme 1) and provides a possible explanation for stabilization of oxygenated intermediates in alkaline electrolytes.

The interaction parameter in alkaline and acidic electrolytes for the catalytically active state Ir^4.x+^ (*O) at the relevant potentials of the OER is significantly different, being 0.35 eV in alkaline and 0.13 eV in acidic electrolytes. The significant weakening of binding energetics of *O species with increasing coverage can be attributed to interactions of these species via the electrolyte. The distance between two *O species, adsorbed on adjacent CUS sites is 3.2 Å, which has been computed to be too large for direct intersite interactions between the adsorbates.^66,67^ In alkaline electrolytes, with increasing coverage, we hypothesize that the solvation of each *O species decreases due to the limited cation concentration that can be present at the interface and the rigid hydrogen-bonding network, which makes solvent restructuring energetically unfavorable. Consequently, the binding energy of the *O intermediates decreases with increasing coverage. Therefore, by directly probing the nature of interfacial water molecules, we propose an interfacial structure that can rationalize the differences in both binding energetics and interaction parameters as a function of pH (Scheme 1).

### 2.4 Implications on the Reaction Kinetics for Water Oxidation

**Figure 5:**
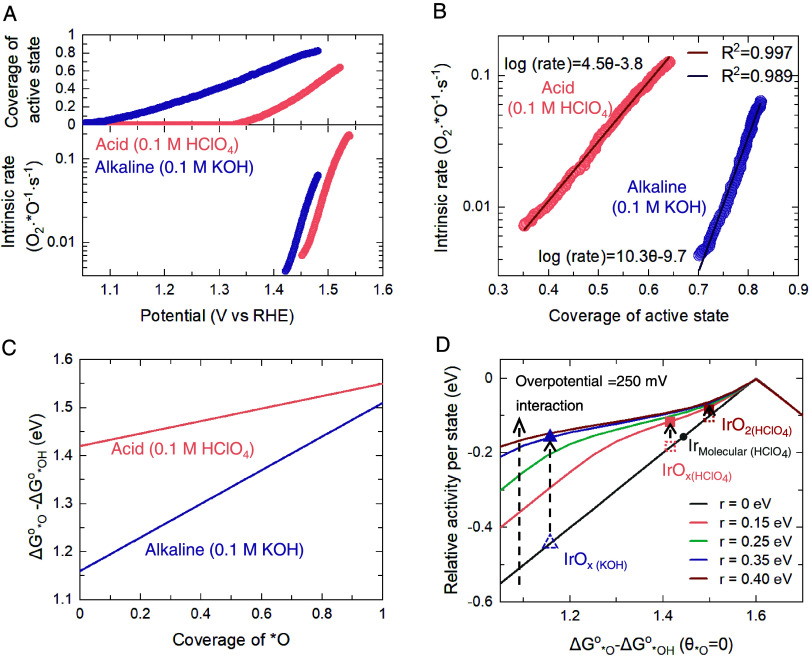
Energetics of active states and their influence on intrinsic water oxidation kinetic. (A) Coverage of active state *O (top panel) and the corresponding intrinsic rate on IrO_*x*_ change as a function of potential in acid and alkaline. (B) Intrinsic rate per active state of IrO_*x*_ as a function of the coverage of *O in 0.1 M HClO_4_ and 0.1 M KOH. (C) Experimentally determined ΔG_*O_^o^*–* ΔG_*OH_^o^ values at different coverages of *O for amorphous IrO_*x*_ in 0.1 M HClO_4_ and 0.1 M KOH, following a Frumkin-isotherm-related equation ΔG_*O_^o^–ΔG_*OH_^o^(θ) *=* ΔG_*O_^o^–ΔG_*OH_^o^(θ_*O_ = 0) + r*θ_*O_, where ΔG_*O_^o^–ΔG_*OH_^o^(θ_*O_ = 0) is the binding energy of *O assuming zero coverage. The values of ΔG_*O_^*o*^–ΔG_*OH_^*o*^ (θ_*O_ = 0) and *r* are determined by fitting the electroadsorption isotherms in Figure 2D,E for alkaline and acid,_,_ respectively. (d) Relative activity per active state at a potential of 1.48 V_RHE_ and the corresponding position of IrO_*x*_ under alkaline conditions and previously reported amorphous, rutile iridium oxides and Ir molecular catalysts under acidic conditions (0.1 M HClO_4_).^42,44^ The relative activity is evaluated by the thermodynamic overpotential, which is calculated as the absolute difference between the energetic ΔG_*O_^*o*^–ΔG_*OH_^*o*^ and the theoretical optimal value of 1.6 eV,^3,4^ i.e., relative activity = −|ΔG_*O_^*o*^–ΔG_*OH_^*o*^ (θ)–1.6 eV|. The coverage of active states was obtained by numerically solving the Frumkin isotherm equation at a given *r* and ΔG_*O_^o^–ΔG_*OH_^o^ (θ_*O_ = 0).

Having quantitatively determined the nature and density of active states and the interfacial solvent environment on IrO_*x*_ as a function of potential (Scheme 1), we next discuss the influence of these on the intrinsic water oxidation kinetics. We estimate the reaction kinetics by dividing the rate of O_2_ generation by the concentration of the active states, assuming a Faradaic efficiency of 100%. This gives the active-state-normalized rate of O_2_ release, which is equivalent to the intrinsic rate of the RDS (denoted as *R*_rds_, with a unit of [O_2_·*O^–1^·s^–1^]). Figure 5A compares the coverage of active states and the intrinsic rate of water oxidation for IrO_*x*_ in acidic and alkaline media as a function of potential. IrO_*x*_ forms active states at around 200 mV lower in acidic than in alkaline electrolytes and has a higher coverage at a given potential in the observed potential regime, which we hypothesize to be a result of the stabilization of *O intermediates by hydrated cations at low coverage. Moreover, the rate of increase in *O coverage with the potential is slower in alkaline compared to acid, while the TOF in both electrolytes increases at a similar rate with the potential. This suggests that the lower Tafel slope of IrO_*x*_ in the alkaline electrolyte shown in Figure 1B, as compared to the acidic electrolyte, results from the difference in the potential dependence of the *O coverage. Figure 5A also shows that at a given potential, IrO_*x*_ shows a comparable or slightly higher intrinsic rate in the alkaline electrolyte compared to the acidic electrolyte (factor of ∼2–3). Notably, an appreciable release of O_2_ (0.005 O_2_. *O^–1^·s^–1^) is observed only in alkaline at an *O coverage of ∼0.7; while in the acidic electrolyte, this level of O_2_ release is observed at a lower coverage of ∼0.3. These results suggest that although the *O species are formed at less positive potentials in alkaline solutions, they are not active for catalyzing the formation of the O–O bond to generate O_2_. These could be explained by the lower energetics of these states in alkaline once formed. As discussed in Sections 2.2 and 2.3, the energetics of the states are a function of their coverage due to the lateral interaction between them. Specifically, the lateral interaction in the case of the alkaline electrolyte is ∼2.7 times larger as compared to the acidic electrolyte. Nong et al. have suggested that the O–O bond formation step on IrO_*x*_ is chemical in nature and is driven by the coverage of *O species.^41^ Therefore, based on this model, with increasing coverage of *O species, the energetics of O–O bond formation linearly decreases, resulting in an Eyring-like equation
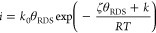
1where the prefactor *k*_0_ is an attempt frequency for the reaction and independent of θ_RDS_, θ_RDS_ is the potential-dependent coverage of the active state (i.e., the state from which the RDS takes place, *O in this study), ζ is the constant to determine the change in O–O bond formation energetic with coverage of active states (proportional to the interaction parameter *r*), *k* is the constant to determine the O–O bond formation energetic at zero coverage of active states, and *R* and *T* are the gas constant and temperature, respectively. A comparison of the intrinsic rates in acid and alkaline as a function of the coverage is shown in Figure 5B. The intrinsic rate for the acidic electrolyte is much higher than that in the alkaline electrolyte at a given coverage. Additionally, we note that the logarithm of intrinsic rate increases linearly with the active state coverage in the observed coverage range, with a slope of ∼4.5 and ∼10.3 for acid and alkaline, respectively. This result is similar to the observation in a recent work by Nong et al.^41^ as well as our own work,^42^ where the logarithm of the current was found to linearly increase with the *O coverage, in agreement with eq 1. Here, we note that the change in slope between the electrolytes is ∼2.3 (10.3 in alkaline versus 4.5 in acid), which is in close agreement with the degree of change of the interaction parameter between the electrolytes (around 0.35 eV in alkaline versus 0.13 eV in acid). This further validates that the weakening of the *O binding with increasing coverage is directly related to the increase in the kinetics of the O–O bond formation.

In order to rationalize the coverage- and potential-dependent activity of IrO_*x*_ in acidic and alkaline electrolytes, we extract the value of (ΔG_*O_^*o*^–ΔG_*OH_^*o*^) as a function of coverage based on the Frumkin electroadsorption isotherm in Figure 2D,E. The Gibbs free energy of active states at zero coverage, i.e., ΔG_*O_^o^–ΔG_*OH_^o^(θ_*O_ = 0) observed in Frumkin fitting, is lower in the case of the alkaline electrolyte (∼1.16 eV) compared to the acidic electrolyte (∼1.42 eV). However, with increasing coverage, the difference in the (ΔG_*O_^o^–ΔG_*OH_^o^) value decreases, owing to the higher interaction parameter in alkaline electrolytes (Figure 5C). Computational studies have demonstrated that IrO_2_ binds *O more strongly than its optimal; thus, weakening the binding energetics of *O should result in higher OER kinetics. We can thus explain the trends in the intrinsic rate as a function of coverage: at low coverages, the higher ΔG_*O_^o^–ΔG_*OH_^o^ or the weaker *O binding energetics in the acidic electrolyte makes the OER more than an order of magnitude faster; this difference becomes less with increasing coverage due to the higher interaction parameter in alkaline electrolyte and consequently the difference in activity between the two electrolytes decreases with increasing coverage. Finally, the intrinsic rate at a given potential is a convolution of the density of active states (higher in the alkaline electrolyte, Figure 5A) and intrinsic rate per active state (higher in the acidic electrolyte, Figure 5B); the opposing trends result in a similar potential-dependent intrinsic rate of reaction.

The conventional volcano plot, which uses ΔG_*O_^o^–ΔG_*OH_^o^ as the descriptor and implicitly assumes that this value is coverage and electrolyte independent, is widely used for predicting catalyst activity. However, our experimental finding, as well as several reports for comparing IrO_*x*_ under acid and alkaline conditions,^9,40^ cannot be explained by this model. Instead, our recent work proposes a new model that incorporates absorbate–absorbate interactions, which affect intermediate energetics and thus reaction barriers.^42^ This new volcano plot has been shown to capture the trend of water oxidation activity between amorphous IrO_*x*_ and crystalline IrO_2_, where the ΔG_*O_^o^–ΔG_*OH_^o^ values are 0.1 eV different, but the interaction parameters are the same, as well as for molecular Ir catalysts, which have negligible intersite interactions.^44^ Here, our results show that the activity trends for IrO_*x*_ in acidic and alkaline electrolyte, which have significantly different ΔG_*O_^o^–ΔG_*OH_^o^ as well as interaction parameters, are also captured very well with this model (Figure 5D). Based on our analysis, the predicted activity of IrO_*x*_ in alkaline conditions is significantly improved compared to what would be determined by the conventional volcano plot (volcano line in black, Figure 5D) due to the presence of absorbate–absorbate interactions that drive the theoretical overpotential closer to optimal levels and make it comparable to that observed in acid conditions (see Figure S24 and our previous work^42^ for details of construction of this volcano plot). Therefore, this study not only highlights the crucial role of absorbate–absorbate interactions in controlling catalytic activity but also provides molecular insights into the role of the interfacial electrolyte structure in facilitating these interactions. Our results thus offer a new perspective on how to tailor the interaction energy to enhance catalytic activity and suggest that optimizing the catalyst–electrolyte interactions could potentially unlock the activity of previously thought to be inactive catalysts.

## 3 Conclusions

In this work, we have elucidated the role of the electrolyte on the adsorption energetics of oxygenated intermediates and the OER kinetics of IrO_*x*_ using a combination of time-resolved *operando* optical spectroscopy, X-ray absorption spectroscopy (XAS), and surface-enhanced infrared absorption spectroscopy (SEIRAS). We have identified and quantified three similar redox transitions on IrO_*x*_ in both acidic and alkaline electrolytes as a function of potential from ∼0.5 V_RHE_ to ∼1.5 V_RHE_. The active state at the OER potentials has a highly oxidized Ir center coordinated with oxo species (Ir^4.x+^–*O), which is involved in the rate-determining step of the O–O bond formation. The oxo species bind ∼0.26 eV stronger in alkaline electrolytes compared to acidic electrolytes but have higher repulsive, adsorbate–adsorbate interactions in the alkaline electrolyte (∼0.35 eV) compared to that in acid (∼0.13 eV). We attribute these to the higher concentration of polar water molecules within the cation hydration shells present in the alkaline electrolyte. These can stabilize *O species strongly at low coverage, but as coverage increases, the stabilization effect becomes weaker due to the decrease in the solvation per *O species. On increasing coverage, the interaction between adsorbates, mediated via the electrolyte, results in weakening of the *O binding energetics, and an increase in OER activity, with this effect being significantly larger in alkaline electrolytes. Therefore, although the *O intermediates bind more strongly than optimal in the alkaline electrolyte, the larger interaction parameter results in significant weakening of *O binding at OER-relevant potentials and comparable activity to the acidic electrolyte. Therefore, through our work, we have unraveled the physical origin of the interaction parameter and demonstrated the critical role of the electrolyte in (de)stabilizing OER intermediates and facilitating long-range interactions between them, both of which are crucial in the design of highly active electrochemical interfaces. These findings open new avenues to tailor interfacial properties to increase catalytic activity at polarized solid–liquid interfaces.

## Data Availability

All data in the study are available from the corresponding authors upon reasonable request. Code for data analysis will be available at open-source Web site https://github.com/Caiwu-L.

## Supporting Information Available

Experimental methods; detailed analysis of optical spectra; Frumkin fitting; detailed analysis of XANES and EXAFS data; and additional SEIRAS spectra (PDF)
