# Supplementary material for: Role of Electrolyte pH on Water Oxidation for Iridium Oxides
Source: J Am Chem Soc. 2024 Mar 25;146(13):8928–38. doi: 10.1021/jacs.3c12011 (PMC10996014; doi:10.1021/jacs.3c12011)
Supplement: Supplementary file 1 — ja3c12011_si_001.pdf [file ja3c12011_si_001.pdf]

Supplementary Information

Role of Electrolyte pH on Water Oxidation for Iridium Oxides

Caiwu Liang,[a] Yu Katayama, [c] Yemin Tao, [a] Asuka Morinaga, [c] Benjamin Moss, [b] Verónica Celorrio,[d] Mary Ryan, [a] Ifan E. L. Stephens,[*a] James R. Durrant,[*b] Reshma R. Rao[*a]

a) Department of Materials, Imperial College London, Exhibition Road, SW72AZ, London, United Kingdom

b) Department of Chemistry, Centre for Processable Electronics, Imperial College London, White City campus, W12 0BZ, London, United Kingdom

c) SANKEN (The Institute of Scientific and Industrial Research), Osaka University, Mihogaoka 8-1, Osaka 567-0047, Ibaraki, Japan

d) Diamond Light Source, Harwell Science and Innovation Campus, Didcot, OX11 0DE, UK

Reshma R. Rao: reshma.rao@imperial.ac.uk

James R. Durrant: j.durrant@imperial.ac.uk

Ifan E.L. Stephens: i.stephens@imperial.ac.uk

Supplementary method

Synthesis of amorphous IrO_x film.

The amorphous IrO_x sample was prepared via electrodeposition, following the same method in our previous work.[1, 2] Briefly, we dissolved 0.2 mmol of $IrCl_3$ hydrate and 1 mmol of oxalic acid dehydrate in 30 mL of water, adjusted the pH to 10 with 5 mmol of K_2CO_3, and increased the volume to 50 mL. After resting the solution for 4 days at 35ºC, we used a three-electrode setup to electrodeposit IrO_x onto a clean fluorine-doped tin oxide (FTO) glass substrate. An anodic current density of 35 µA/cm^2 was applied for ~1000s, resulting in a mass loading of around 80 µg/cm^2.

Electrochemical measurement

The electrochemical measurements were carried out in 0.1 M $HClO_4$ and 0.1 M KOH prepared by dilution of concentrated perchloric acid and potassium hydroxide (Suprapur 70% $HClO_4$, and Suprapur 95% KOH, Merck, Germany) in ultrapure water (>18.2 MΩ cm, Sartorius). Iridium oxides samples were deposited on ~1cm x 1cm area of FTO substrates. The electrochemical test was conducted in a typical three-electrode setup using an SP-150 Biologic potentiostat. The catalysts on FTO substrate were used directly as working electrode and test in 0.1 M perchloric acid. A Pt mesh were used as

counter electrodes. A home-made Reversible Hydrogen Electrode (RHE) was used as reference electrode. The RHE electrode is made by sealing a Pt wire in a piece of glass tube with a small open hole at the end act as capillary. The hydrogen is filled using in-situ hydrogen evolution on the Pt wire under the same electrolyte of electrochemical measurement inside the glass tube, *i.e.* 0.1 M $HClO_4$ and 0.1 M KOH, for measurement under acid and alkaline, respectively. Potentials were iR compensated by manually subtracting $I \times R_u$. The resistance R_u was obtained from high-frequency intercept of the real resistance in electrochemical impedance spectroscopy (EIS).

Operando optical spectroscopy measurement

We performed Operando optical spectroscopy measurements on IrO_x on ~1cm x 1cm areas of FTO substrates. The measurements were conducted in a home-made three-electrode cell using a custom-built optical spectroscopy setup. A stabilized 10mW tungsten-halogen light source (Thorlabs, SLS201L) equipped with a collimating add-on (Thorlabs, SLS201C) was used to illuminate the sample. The transmitted light was collected using a 1 cm diameter liquid light guide (Edmund Optics) and sent to a spectrometer (Andor Kymera 193i) equipped with a CCD camera (Andor iDus Du420A-BEX2-DD). To optimize the optical performance of the spectrometer, the light was first collimated and then refocused using two 5 cm plano-convex lenses (Edmund Optics). The CCD camera was cooled to -80°C during the measurements to ensure a high signal-to-noise ratio. The Ivium Vertex potentiostat was used to control the potential, and a custom-built LabView software was developed to facilitate data acquisition. The measurements were performed in potentiostatic mode, and the equilibration time at every 1 mV step was around 1 second. Following equilibration, the optical spectra were acquired by taking 30 spectral averages (each spectral acquisition takes ~30 ms) before moving to the next potential. Simultaneously, the current was measured at each potential using the Ivium Vertex potentiostat.

Operando X-ray absorption spectroscopy

X-ray absorption spectroscopy data of Ir L_3 edge were collected at the B18 beamline at the Diamond light source in UK. The beamline has an energy range of 2.05 keV – 35 keV for spectroscopy. The energy of the incident X-ray beam was selected using a Si (111) monochromator, $\lambda/\Delta\lambda \sim 5{,}000$. The XAS measurement for standard sample Ir powder, $IrCl_3$, and IrO_2 (Alfa Sigma) were perform in transmission mode. The XAS measurements for amorphous IrO_x were performed in fluorescence mode. In both transmission and fluorescence modes a piece of Pt foil was placed between the second and third ionization chambers in order to calibrate the energy to the Pt L_3 edge at 11564 eV. The acquired spectra were extracted, calibrated and normalized using the open source software Athena.[3] The white line (WL) positions are determined by the maximum absorption of the peaks. The WL integration follows a procedure reported by Nataša et. al.[4] The EXAFS spectra were analyzed with open source software

Artemis. The spectra are fitted over a k-range of 3 to 11 $Å^{-1}$and a rutile IrO_2 model (card 81028 ICSD) was used to fit the first scattering path (corresponding to Ir-O).

Operando measurements were performed using a home-built cell that enables us to measure the electrodeposited film on FTO, the same type of sample as used in the operando optical measurement. The cell design (Fig S10) is also able to accommodate electrodes with various substrate and size, as well as electrolyte volume. All the measurements were performed under the same conditions as describe in section Electrochemical Measurement. The electrochemistry in this home-made in-situ cell is identical to normal standard three electrode cell. Before XAS measurement, the electrodes were first cycled using cyclic voltammograms (CVs) at 10 $mV\cdot s^{-1}$ between 0.6 V to 1.45 V_{RHE}. XAS were acquired while performing potential holds at a range of ~0.4 and ~1.50 V_{RHE} (in increasing potential order and come back to the first applied potential to ensure the reversibility). At each potential hold, once the current stabilized, we collected 10 XAS spectra. The final XAS spectra were derived from the average of these 10 collected spectra.

Operando surface-enhanced infrared absorption spectroscopy (SEIRAS)

Pt was first deposited on the total reflecting plane of a hemispherical Si prism (radius 22 mm, Pier optics, Japan) via an electroless deposition method described elsewhere.[5-7] In short, the surface of the Si hemisphere was given a hydrophilic treatment by contacting it with a 40% NH_4F solution for a minute, followed by the deposition of palladium seeds on the base plane with 1% HF–1 mM $PdCl_2$ for 5 min at room temperature. After rinsing with water, platinum electroless deposition was carried out by contacting the Si sphere with the Pt plating solution at 50 °C for *ca.* 12 minutes. The amorphous IrO_x was electrodeposited on the Pt surface, following the same method in the previous section. The CVs of the resultant surface show the characteristic redox peaks from amorphous IrO_x, indicating a negligible effect of Pt underlayer (Fig. S18). The prism was then mounted in a spectroelectrochemical three-electrode cell. An Ag/AgCl reference electrode and a graphite counter electrode were used. The SEIRA spectra were obtained on a Nicolet iS50 (Thermo Fischer Scientific) equipped with a liquid-nitrogen-cooled Mercury Cadmium Telluride (MCT) detector. N_2 was used to fully replace the optical path. The resolution for the measurements was 8 cm^{-1}. For each condition, 32 scans were averaged. The SEIRA spectra were recorded using a custom-made single reflection ATR accessory (Thermo Fischer Scientific) with a Si prism at an incident angle of 67°. All spectra are shown in absorbance units defined as $\log(I_0/I)$, where I_0 and I represent the spectra at the reference and sample potentials, respectively. The reference spectrum I_0 was measured at 0.4 V_{RHE} and 0.5 V_{RHE} in the 0.1 M KOH and 0.1 M $HClO_4$ solution, respectively (Fig. S19 and 20). We note that the same amorphous IrO_x surface was used to measure spectra both in acid and alkaline conditions to ensure a similar surface enhancement effect.

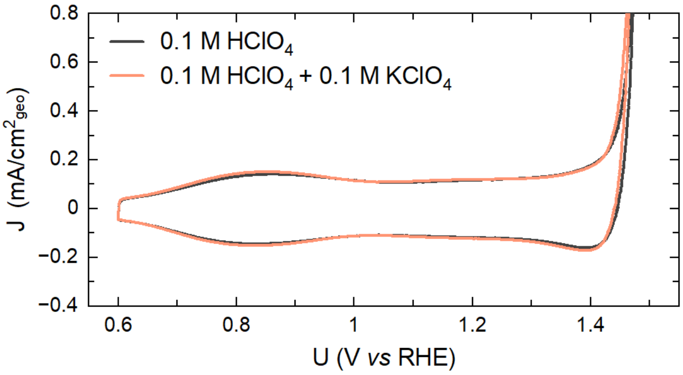

Figure S1 Comparison of cyclic voltammogram of IrO_x in 0.1 M $HClO_4$ + 0.1 M $KClO_4$ electrolyte and in 0.1 M $HClO_4$ at a scan rate of 10 mV s^{-1} at room temperature, with iR corrected manually using high frequency resistance measured in electrochemical impedance spectroscopy. The redox transition peak positions in CV at electrolyte with K^+ cation is similar with that of electrolyte without K^+, indicating the significant shift of redox peak position in 0.1 M KOH compared with 0.1 M $HClO_4$ is not an effect of cation.

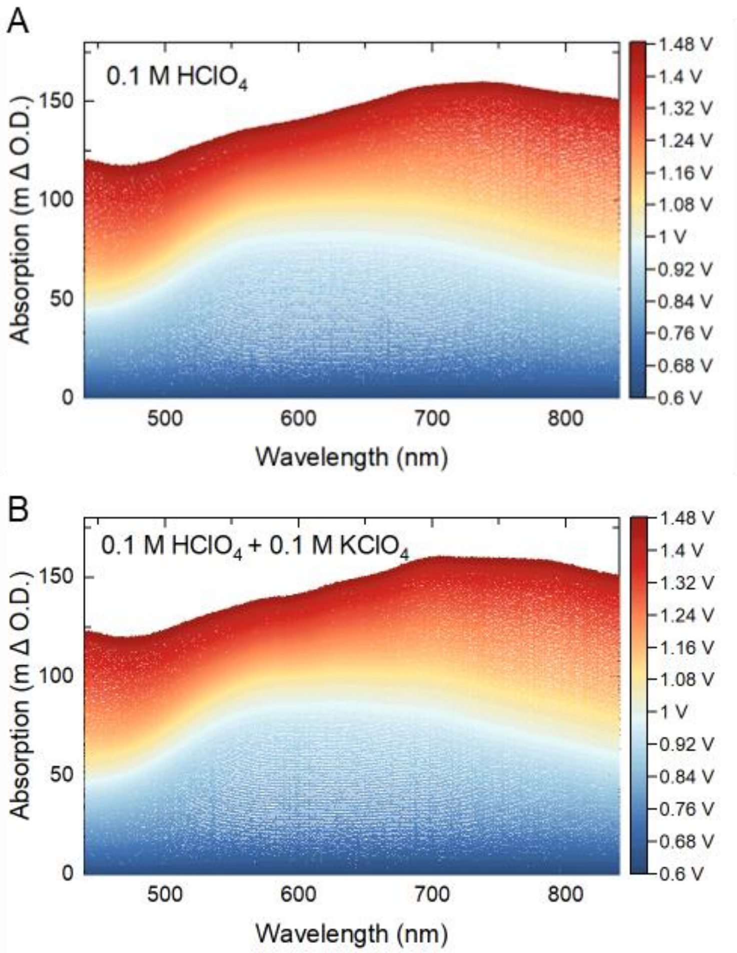

Figure S2 Comparison of optical absorption spectra in 0.1 M $HClO_4$ without (A) and with (B) adding 0.1 M $KClO_4$. The absorption spectra are measured during a linear sweep scan from 0.6 V to ~1.482 V_{RHE}, at a scan rate of 5 mV s^{-1} (iR corrected). Absorption changes were recorded at every 5 mV. The absorption changes are calculated with respect to the absorption at 0.6 V_{RHE}.

Supplementary note 1

Deconvolution of optical absorption spectroscopy

The method and programming scripts for deconvolution of spectra are first reported in our previous work.[2] Briefly, the deconvolution procedure includes three steps: 1) Obtain individual absorption spectra for species by differential analysis; 2) Perform linear combination fitting for spectra obtained at all the measured potentials to get individual contribution from each specie. 3) Experimentally measuring extinction coefficient of each species and converting absorption to density of species using Beer-Lambert law. The detailed process of each step is reported in our previous work.[2] The detailed spectra and deconvolution results in acid and alkaline in this study are as follows:

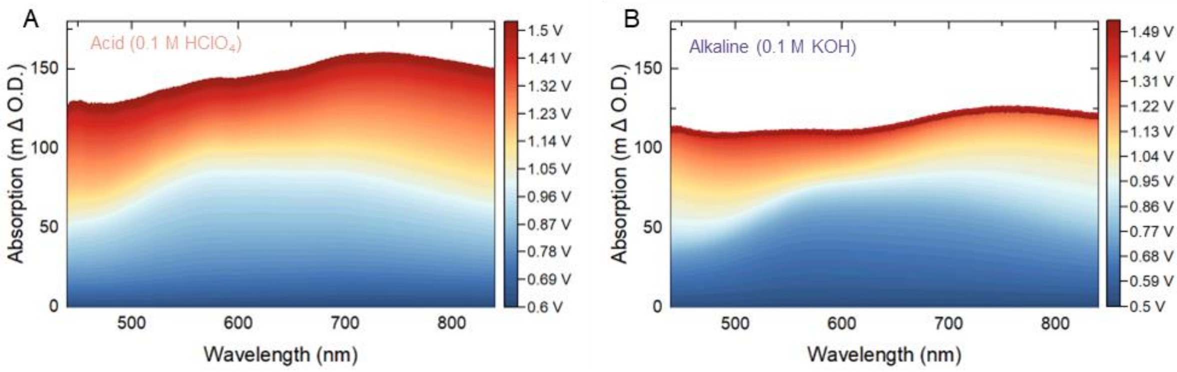

Figure S3 Absorption spectra of amorphous IrO_x in 0.1 M HClO4 (A) 0.1 M KOH (B) during a linear sweep scan from 0.6 V to 1.538 V_{RHE} and 0.5 to 1.535 V_{RHE}, respectively, at a scan rate of 1 mV s^{-1} (iR corrected). Absorption changes were recorded at every 1 mV. The absorption changes are calculated with respect to the absorption at 0.6 V and 0.5 V_{RHE} for acid and alkaline respectively.

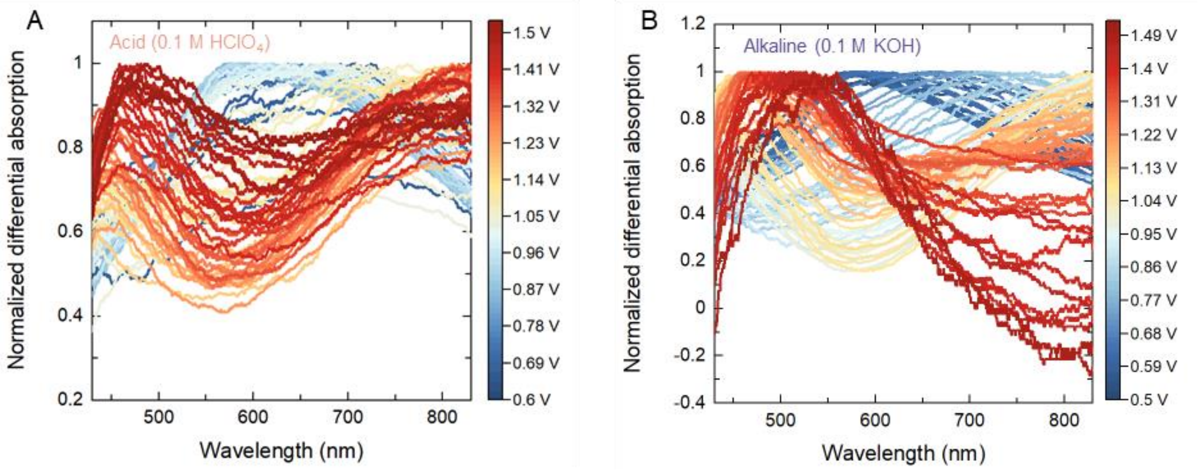

Figure S4 (A) Differential analysis of absorption spectra for IrO_x in acid. Differential absorption at every 10 mV. The absorption was obtained by subtraction of adjacent 10 mV spectra and normalized the maximum absorption to 1. The individual spectra of redox transitions were extracted at potential regime where spectra doesn't change obviously in shape, which corresponds to a change of the density of individual species. The absorption of redox transition 1, 2 and 3 were extracted from the differential absorption between 0.91-0.90, 1.23-1.22 and 1.54-1.47 V, respectively. (B) Differential analysis of absorption spectra for IrO_x in alkaline at every 10 mV. The absorption of redox transition 1, 2 and 3 were calculated by the differential absorption between 0.68-0.67, 1.06-1.05 and 1.41-1.40 V, respectively.

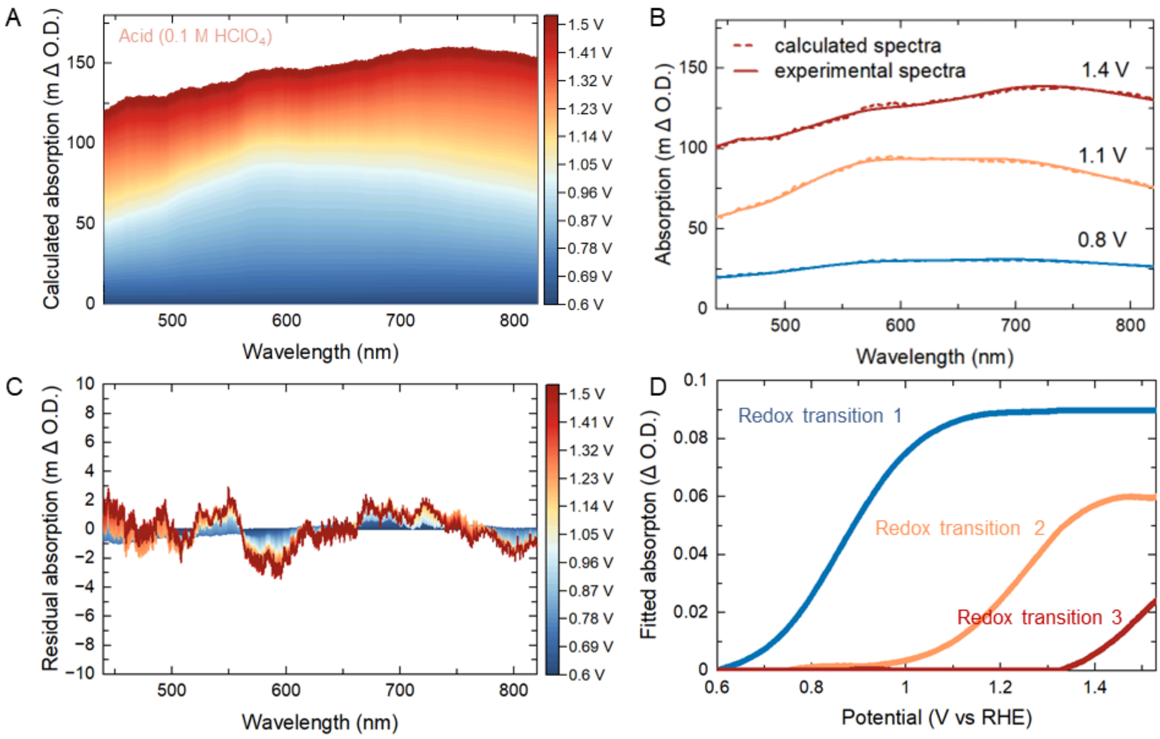

Figure S5. Deconvolution result for IrO_x in acid. (A) Calculated differential absorption spectra of IrO_x in acid at every 1 mV during a linear sweep scan from 0.6 V to 1.538 V_{RHE}. (B) Comparison of calculated differential spectra and experimentally observed spectra. (C) Fitting residuals between calculated spectra and experimental spectra at each potential. (D) Calculated absorption at its peak wavelength of each redox transition as a function of potential.

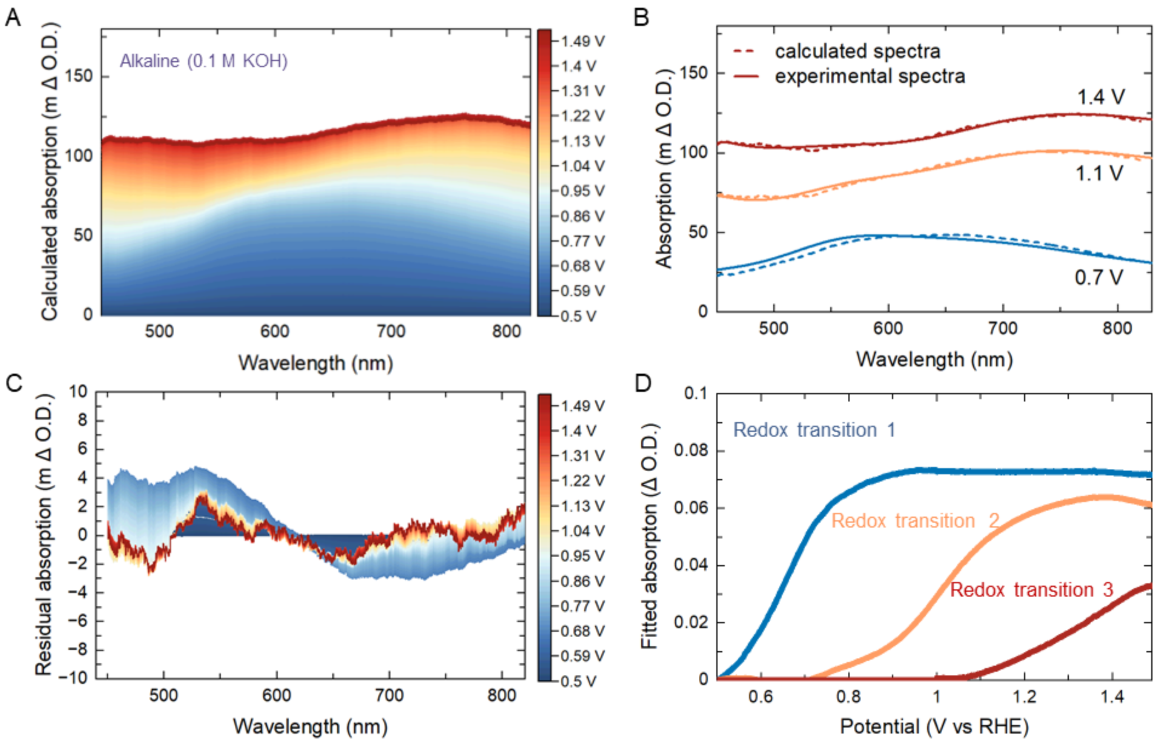

Figure S6. Deconvolution result for IrO_x in alkaline. (A) Calculated differential absorption spectra of IrO_x in alkaline at every 1 mV during a linear sweep scan from 0.5 to 1.535 V_{RHE}. (B) Comparison of calculated differential spectra and experimentally observed spectra. (C) Fitting residuals between calculated spectra and experimental spectra at each potential. (D) Calculated absorption at its peak wavelength of each redox transition as a function of potential.

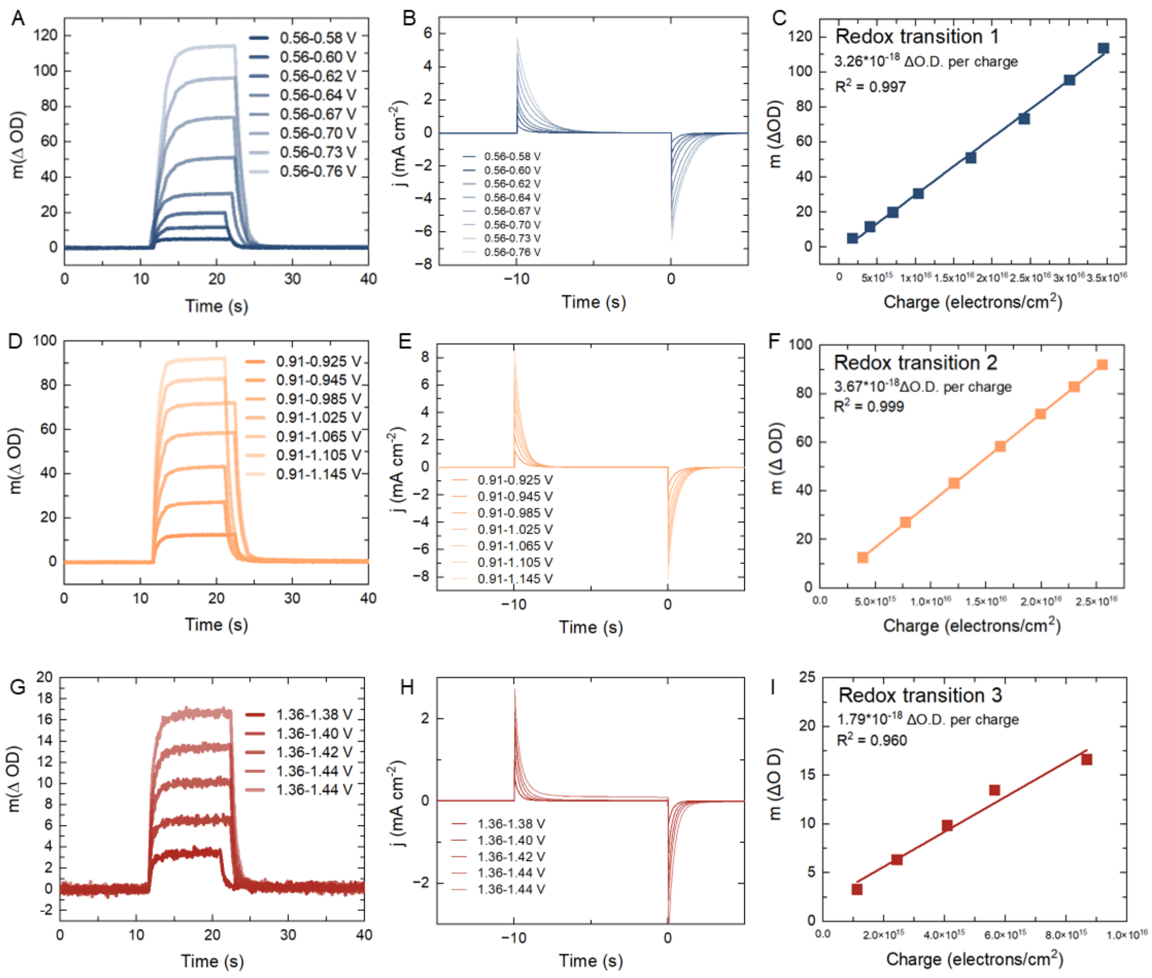

Figure S7. Measurement of extinction coefficient $\varepsilon_i(\lambda)$ for each redox transition in IrO_x under alkaline. (A) Absorption changes at 600 nm during potential steps between two different applied potentials. (B) the corresponding transient currents measured during potential (C) The relation between deconvoluted absorption change and the extracted charge for redox transition 1. The charges are calculated by integrating the reductive peak in B. The slope of the linear regression is the differential absorption coefficient of the redox transition 1. (D-F) Absorption changes at 800 nm, current and charge vs absorption relationship for redox transition 2. (G-H) Absorption changes at 500 nm, current and charge vs absorption relationship for redox transition 3.

Supplementary Note 2

Since the structure of amorphous IrO_x is not well defined, to estimate its Ir density per volume we assume a hollandite structure which has larger channel sizes in the structure that possibly resembles the porous structure of amorphous IrO_x.[8] According to the lattice parameters of this structure, the volume density of Ir site is around ~6.35 Ir/nm^3_{oxide}. To evaluate how much oxide is present per geometric area of electrode surface, we consider the simplest model of iridium oxide distribution: (1) the nanoparticles are in ideal and smooth half sphere shape with radius of *r* and (2) are densely packed on the electrode surface (as schematically illustrated in Fig. S8). Thus, the volume of iridium oxide per electrode surface area are:

$$V = \frac{\frac{1}{2}\times\frac{4}{3}\pi r^3}{(2r)^2} = \frac{\pi r}{6}\ (\mathrm{nm^3_{oxide}/nm^2_{substrate}})$$

In this case we have Ir site density *D* as:

$$\mathrm{D} = \mathrm{V} \times 6.35 = (6.35\,\pi r)/6\ \ (\mathrm{Ir}/nm^2)$$

Given that the nanoparticle size is around 100-200 nm, assume the mean particle size of 150 nm, resulting in *r* = 75 nm. This results in a Ir site density of ~ 2.49 $\times10^{16}$ Ir/cm^2. As shown in Fig 2 A in the main manuscript, the density of Ir that participate in *redox transition 1* is around 2.75$\times10^{16}$ Ir/cm^2. Although the models used for evaluation are significantly simplified and contain several assumptions as stated above, the results suggest that the number of active Ir sites measured are in the same order of the total Ir sites in the nanoparticle in a simple model. This could explained by the highly porous structure of amorphous IrO_x and is consistent with works by Dau et al that shows amorphous cobalt oxides exhibits typical volume activity.[9 10, 11]This is also in agreement with our previous work that shows amorphous IrO_x has around one order high redox active centres compared with rutile IrO_2.[2]

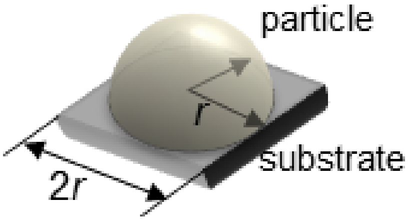

Figure S8. Schematic showing the simplest model of iridium oxide distribution: (1) the nanoparticles are in ideal and smooth half spheres with radius of *r* and (2) are densely packed on the electrode surface.

Supplementary Table S1. Comparison of extinction coefficient of IrO_x in acid and alkaline

Sample	Coefficient Redox transition 1 (OD charge^{-1} cm^{-2})	Coefficient Redox transition 2 (OD charge^{-1} cm^{-2})	Coefficient Redox transition 3 (OD charge^{-1} cm^{-2})
IrO_x (acid)	3.27×10^{-18}	2.23×10^{-18}	1.27×10^{-18}
IrO_x (alkaline)	3.27×10^{-18}	3.67×10^{-18}	1.79×10^{-18}

Extinction coefficient measurement of IrO_x in acid are obtained from our previous work[2]

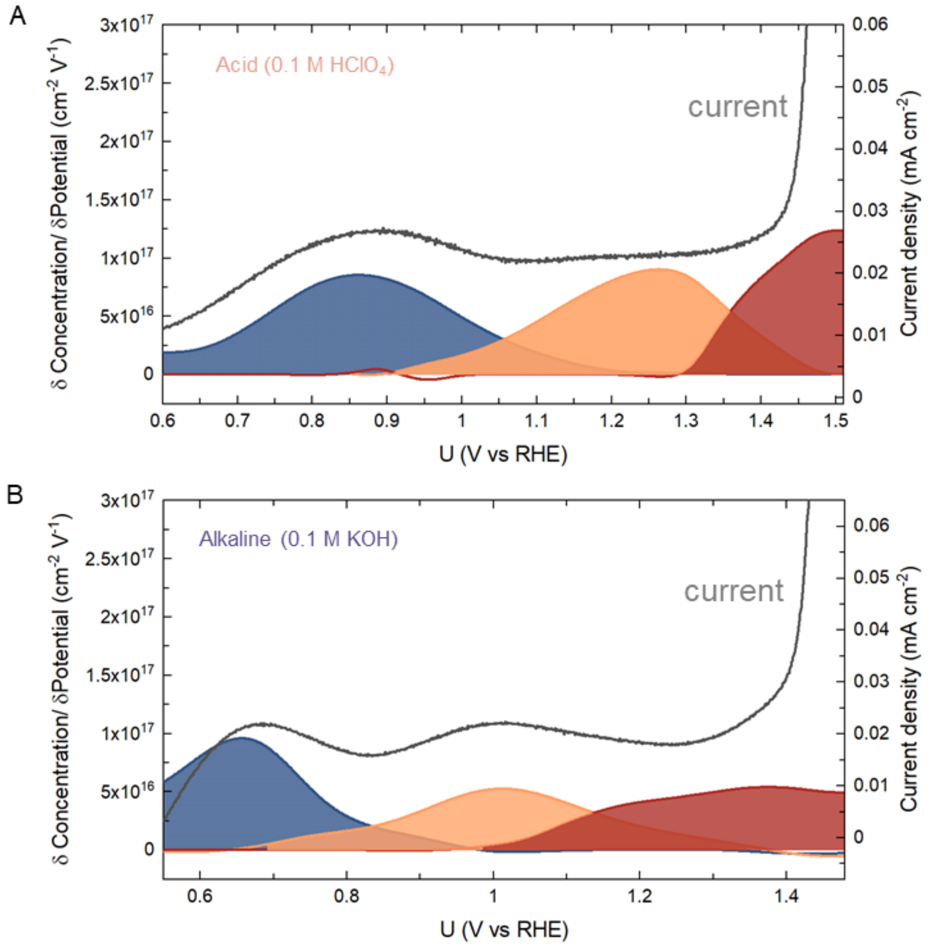

Figure S9. Redox transition waves obtained from operando optical absorption and the corresponding linear scan voltammetry curves (grey light, right-y axis) during measurement for IrO_x under acid condition (A) and alkaline conditions (B).

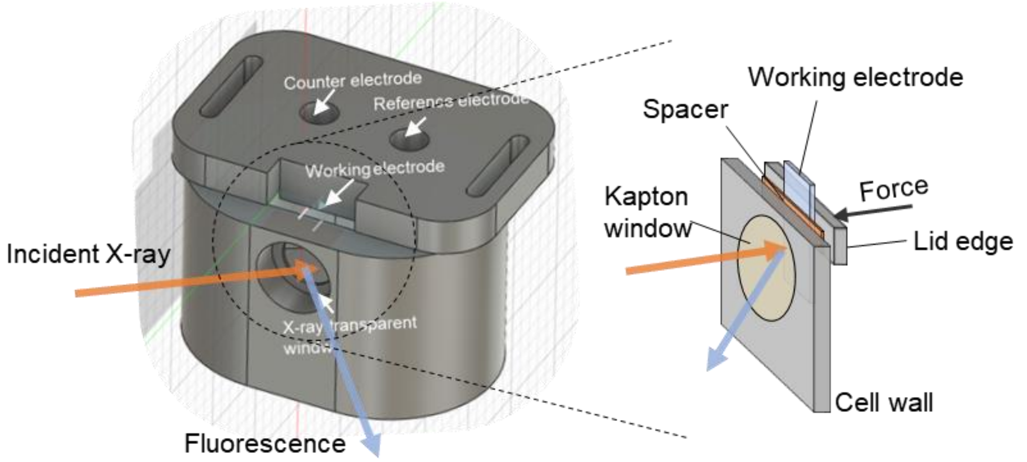

Figure S10. Schematic of in-situ XAS cell. Kapton foil is used as window to minimize the attenuation of X-ray. Schematic showing working principle of the home-built operando XAS cell. Working electrodes (IrO_x film on FTO) are sandwiched between the cell's lid edge and the wall with Kapton X-ray transparent window. A spacer is positioned between the cell wall and the sample to maintain a appropriate distance between the sample and the window.

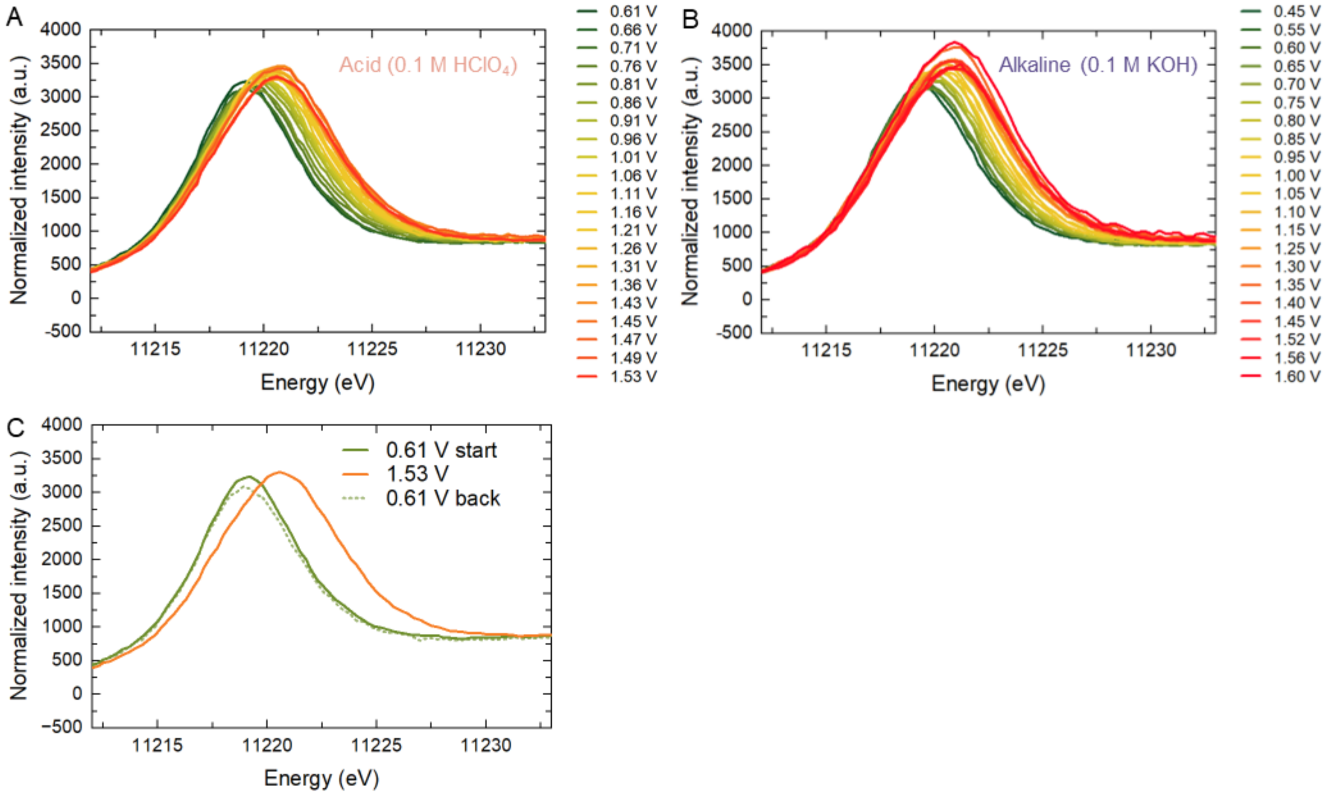

Figure S11. Ir L_3-edge XANES region of IrO_x on FTO measured at different potentials in acid(A) and alkaline (B). (C) Comparison of XANES measured at 0.61 V in acid before and after a series of measurements ranging from 0.61 to 1.53 V_{RHE}, and subsequent return to 0.61 V. The identical WL peak positions at 0.61 V in both spectra indicate the stability and reversibility of the IrO_x sample under XAS measurement.

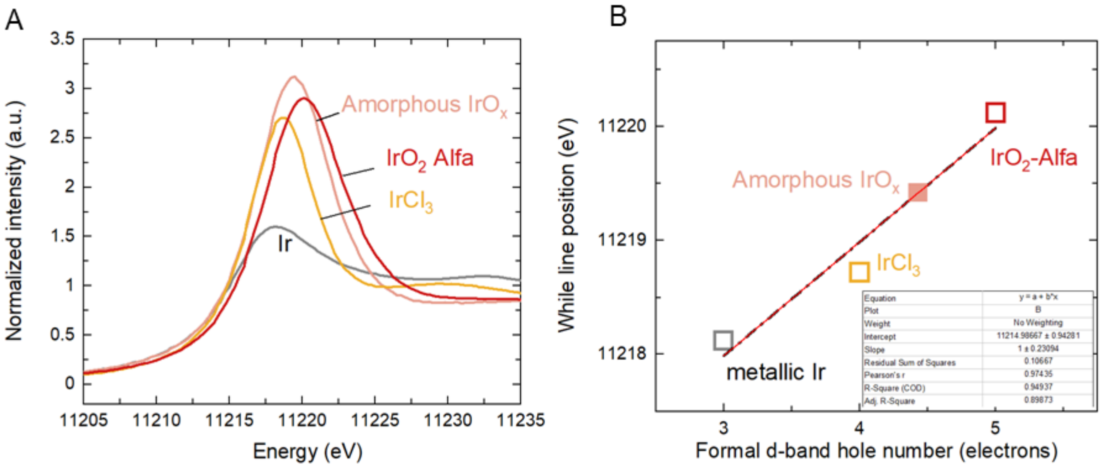

Figure S12. (A) Ir L_3-edge XANES region of IrO_x and the reference standard samples (B) White line position of IrO_x and Ir standard reference as a function of the formal *d*-band hole number. The formal *d*-band hole number for metallic iridium ($5d^7$), $IrCl_3$ ($5d^6$) and IrO_2 ($5d^5$) were 3, 4 and 5 respectively. The white line shift per *d*-band hole (slope) was fitted to be around 1 eV per *d*-band hole. These figures are adapted from our previous work.[2] These reference data and all the XAS data reported in this work were measured in the same period of time and at the same beamline (B17) at Diamond Light Source.

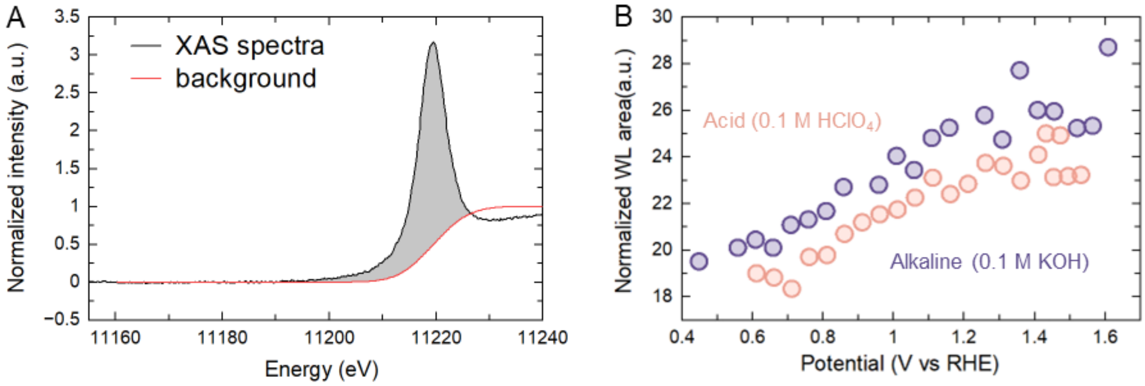

Figure S13 (A) An example of integral area of Ir L_3 white line analysis of IrO_x in alkaline at 0.45 V_{RHE}. The background was modeled using a cumulative Gaussian distribution arctangent function.[4] The black lines show the experimental spectra and the red lines show the background curves and the grey area is the integral area of Ir L_3 white line. Detailed MATLAB analysis scripts is attached in Data availability section. (B) The Ir L_3 white line area of IrO_x as a function of applied potential in acid and alkaline conditions.

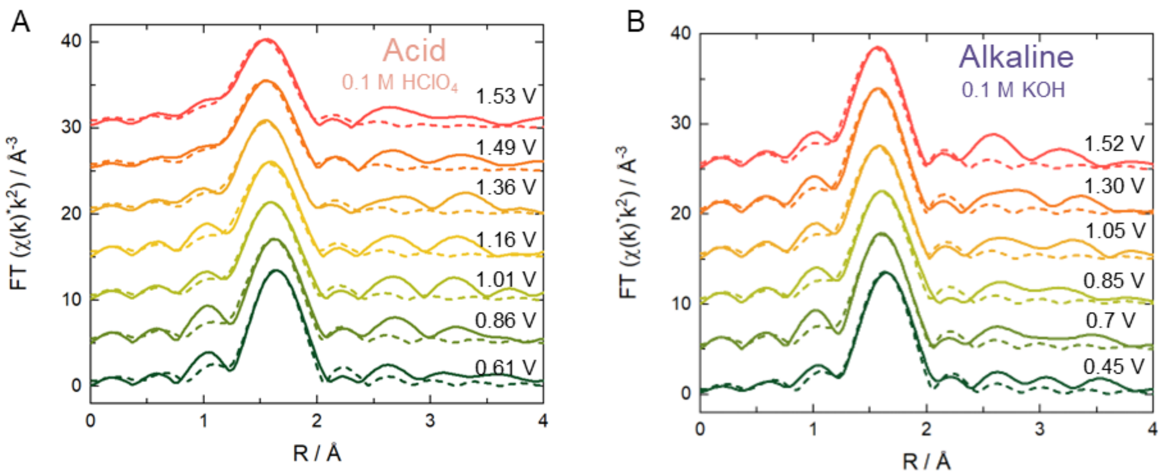

Figure S14 k^2-weighted Fourier transforms of EXAFS spectra collected at the iridium L3 edge of the IrO_x in 0.1M $HClO_4$ (A) and in 0.1M KOH (B). Experimental data (solid lines) and fits (dotted lines) are shown. Detail fitting results are shown in Table S2

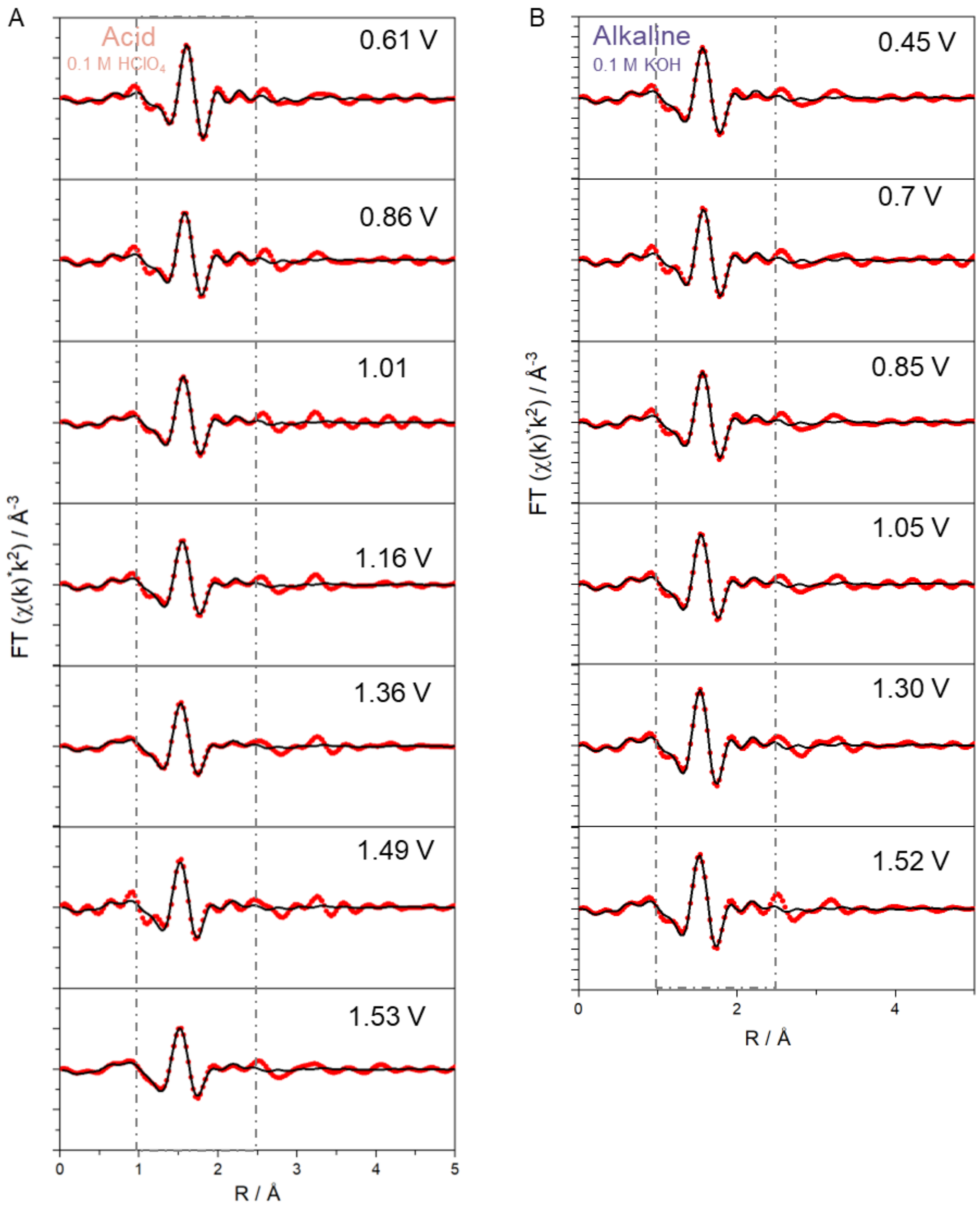

Figure S15 k^2-weighted Fourier transforms of EXAFS spectra collected at the Ir L_3 edge of IrO_x in acid (A) and alkaline (B). The labelled potential are in RHE and Ir corrected. The experimental (red symbols) and fits (black solid lines) are shown.

Supplementary Table S2. Ir L3 edge EXAFS simulation parameters of IrO_x in acid and alkaline at different applied potential. K = 3 – 11 $Å^{-1}$

Sample	Shell	N	σ^2 / $Å^2$	R / Å	ΔE_0 / Ev	R-factor (%)
Acid-0.61 V	Ir-O	6.25 (± 0.71)	0.0033 (± 0.0015)	2.04 (±0.01)	9.34 (± 1.36)	1.2
Acid-0.86 V	Ir-O	6.00 (± 0.82)	0.0047(± 0.0018)	2.02 (±0.01)	9.43 (± 1.55)	1.5
Acid-1.01 V	Ir-O	6.36 (± 0.62)	0.0056 (± 0.0014)	2.00 (±0.01)	8.48(± 1.18)	0.8
Acid-1.16V	Ir-O	6.60 (± 0.81)	0.0066 (± 0.0019)	1.99 (±0.01)	8.16(± 1.47)	1.3
Acid-1.36 V	Ir-O	6.47 (± 0.53)	0.0068 (± 0.0012)	1.97 (±0.01)	7.20 (± 0.97)	0.5
Acid-1.49 V	Ir-O	6.70(± 0.73)	0.0074 (± 0.0017)	1.96 (±0.01)	6.88(± 1.33)	0.9
Acid-1.53 V	Ir-O	6.96 (± 0.72)	0.0081 (± 0.0017)	1.96 (±0.01)	6.78(± 1.25)	0.8
Acid-0.61 V (reverse)	Ir-O	5.99(± 0.86)	0.0027 (± 0.0018)	2.04 (± 0.01)	9.30 (± 1.73)	2.2
Alkaline-0.45 V	Ir-O	5.85 (± 0.61)	0.0027 (± 0.0013)	2.04 (± 0.01)	9.26 (± 1.25)	0.9
Alkaline-0.7 V	Ir-O	6.35(± 0.68)	0.0044 (± 0.0015)	2.02 (± 0.01)	8.77 (± 1.31)	0.9
Alkaline-0.85 V	Ir-O	6.85(± 0.85)	0.0054 (± 0.0019)	2.01 (± 0.01)	9.07 (± 1.48)	1.4
Alkaline-1.05 V	Ir-O	6.74(± 0.66)	0.0055 (± 0.0014)	1.98 (± 0.01)	7.90 (± 1.21)	0.8
Alkaline-1.30 V	Ir-O	6.57(± 0.77)	0.0044 (± 0.0016)	1.97 (± 0.01)	7.84 (± 1.44)	1.1
Alkaline-1.52 V	Ir-O	6.66(± 0.81)	0.0049 (± 0.0017)	1.96 (± 0.01)	7.83 (± 1.49)	1.2

Debye-Waller parameters (σ), coordination distances (R) and energy shift (ΔE_0) were kept unrestricted. Coordination numbers (N) was kept unrestricted for IrO_x. The passive electron reduction factor $S_0{}^2$ was set to 1 to fit the coordination number since the absolute coordination number for Ir in amorphous IrO_x is not known due to the disordered structure. To note, the assumption of $S_0{}^2 = 1$ is made solely to fix the value of $S_0{}^2$, allowing us to observe the changes in the coordination number with potential. The absolute value of the coordination number at a given potential may vary by approximately 20% due to the fitting error bar and the fact that $S_0{}^2$ value are usually below 1. We also note that the determination of coordination distances R is independent of the assumption of $S_0{}^2$ value.

Supplementary note 3: Frumkin isotherm fitting

We model the free energies of adsorbates on surfaces during redox transitions using the following equation, as reported in our previous work[2]:

$$\Delta G = \Delta G^0 + RT\ln(\theta/1-\theta) \qquad (\textit{Equation 1})$$

where θ represents coverage, T is temperature, and R is the universal gas constant. ΔG^o denotes the intrinsic chemical potential for each redox transition. Coverage, θ, is calculated as the ratio of current to maximum density ($\theta = D/D_{max}$), derived from our spectroelectrochemical analyses. Substituting the $\Delta G = -nFU$ and $\Delta G^o = -nFU^o$ into Equation 1 and refer potential to reversible hydrogen electrode, we have Langmuir-type electroadsorption isotherm:

$$U = U^o - \frac{RT}{nF} RT\ln(\theta/1-\theta) \qquad (\textit{Equation 2})$$

We fitted the Langmuir and Frumkin isotherm models to the coverage vs U data obtained by spectroelectrochemistry, which assumes a constant ΔG_0 value in *Equation 1*. As shown in Fig. S16 A and S17 A, the Langmuir isotherm provides an inadequate fit for all the redox transition for IrO_x in both acid and alkaline conditions. The poor fit provided by the Langmuir isotherm model suggests non-negligible adsorbate-adsorbate interactions. To better represent these interactions, we adopt the Frumkin isotherm model, which use a mean-field approximation to to capture the adsorbate–adsorbate interaction.

$$\Delta G^0_{(\theta)} = \Delta G^0_{(\theta=0)} + r \cdot \theta \qquad (\textit{Equation 3})$$

Integrating these concepts, we have Frumkin-type electroadsorption isotherm:

$$U_{RHE} = U^o_{RHE(\theta=0)} + \frac{RT}{nF} RT\ln(\theta/1-\theta) + \frac{r}{nF} \cdot \theta \qquad (\textit{Equation 4})$$

Where r is the lateral interaction parameter represent the interaction strength between the adsorbates, $U^o_{RHE(\theta=0)}$ is the potential of reaction assuming no coverage. Positive value of r corresponds to repulsive interactions, while negative corresponding to attractive interactions. In such Frumkin isotherm, the value of r and $U^o_{RHE(\theta=0)}$ can be obtained by fitting the U vs θ data using *Equation 4*, while the potential of mid-wave ($U_{(\theta=1/2)}$) of redox transition can be calculated as $U_{(\theta=1/2)} = U^o_{RHE(\theta=0)} + 1/2 \frac{r}{nF}$. Fig. S16 B and S17 B show the fit based on the Frumkin isotherm. The fitted values of $U_{(\theta=1/2)}$ and r are labelled in Fig. 2D and 2E in main text. To note, we do not include the very initial or final regime coverage <0.05 or >0.95 considering the relative less confidence in the deconvolution procedure due to the noise in optical signal. The Frumkin isotherm fit match well with the experiment, giving R^2 up to 0.99 in each redox transition of IrO_x in both acid and alkaline condition.

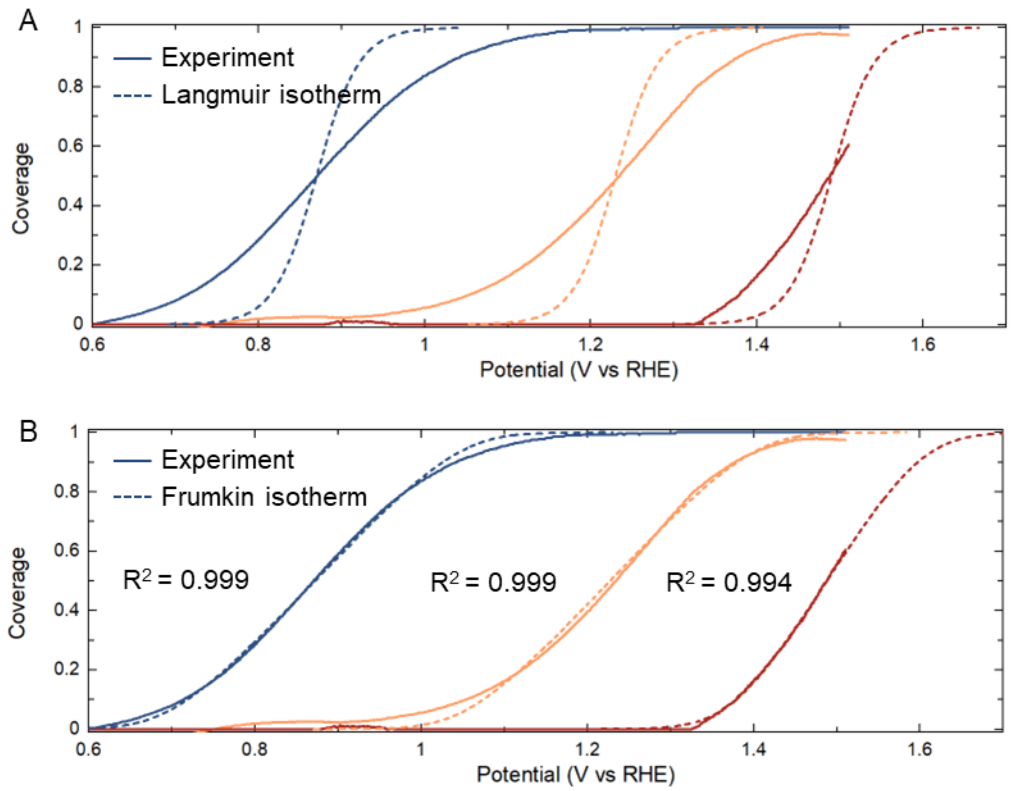

Figure S16. Electro-adsorption isotherm model for IrO_x in aicd (A) Langmuir isotherm fitting for each redox transition of IrO_x under acid condition. Clearly, Langmuir isotherm doesn't fit our experiment data. Frumkin isotherm fitting for each redox transition of IrO_x in acid. This fitting gives R^2 as high as 0.994-0.999. The coverage are fitted in the coverage regime of ~0.05 to ~0.95.

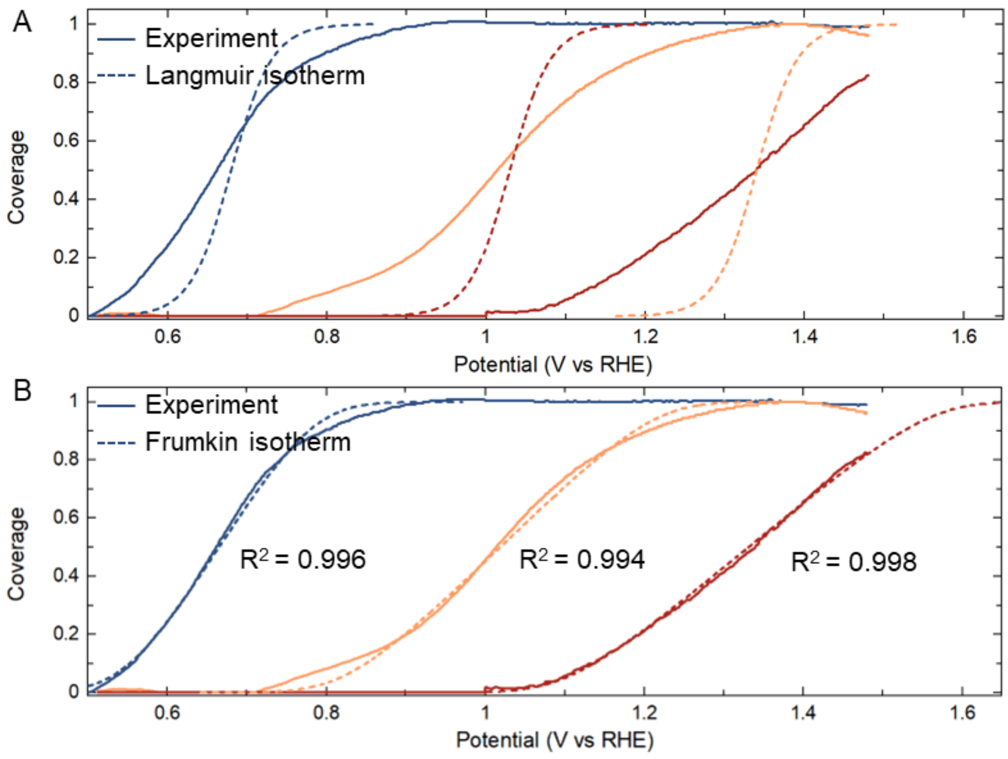

Figure S17. Electro-adsorption isotherm model for IrO_x in alkaline (A) Langmuir isotherm fitting for each redox transition of IrO_x under alkaline condition. Clearly, Langmuir isotherm doesn't fit our experiment data. Frumkin isotherm fitting for each redox transition of IrO_x in alkaline. This fitting gives R^2 as high as 0.994-0.998. The coverage are fitted in the coverage regime of ~0.05 to ~0.95.

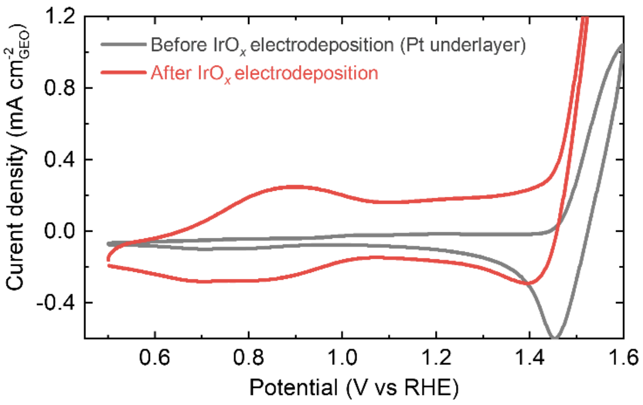

Figure S18 CV comparing SEIRA surface before (Pt) and after the IrO_x electrodeosition in $HClO_4$.

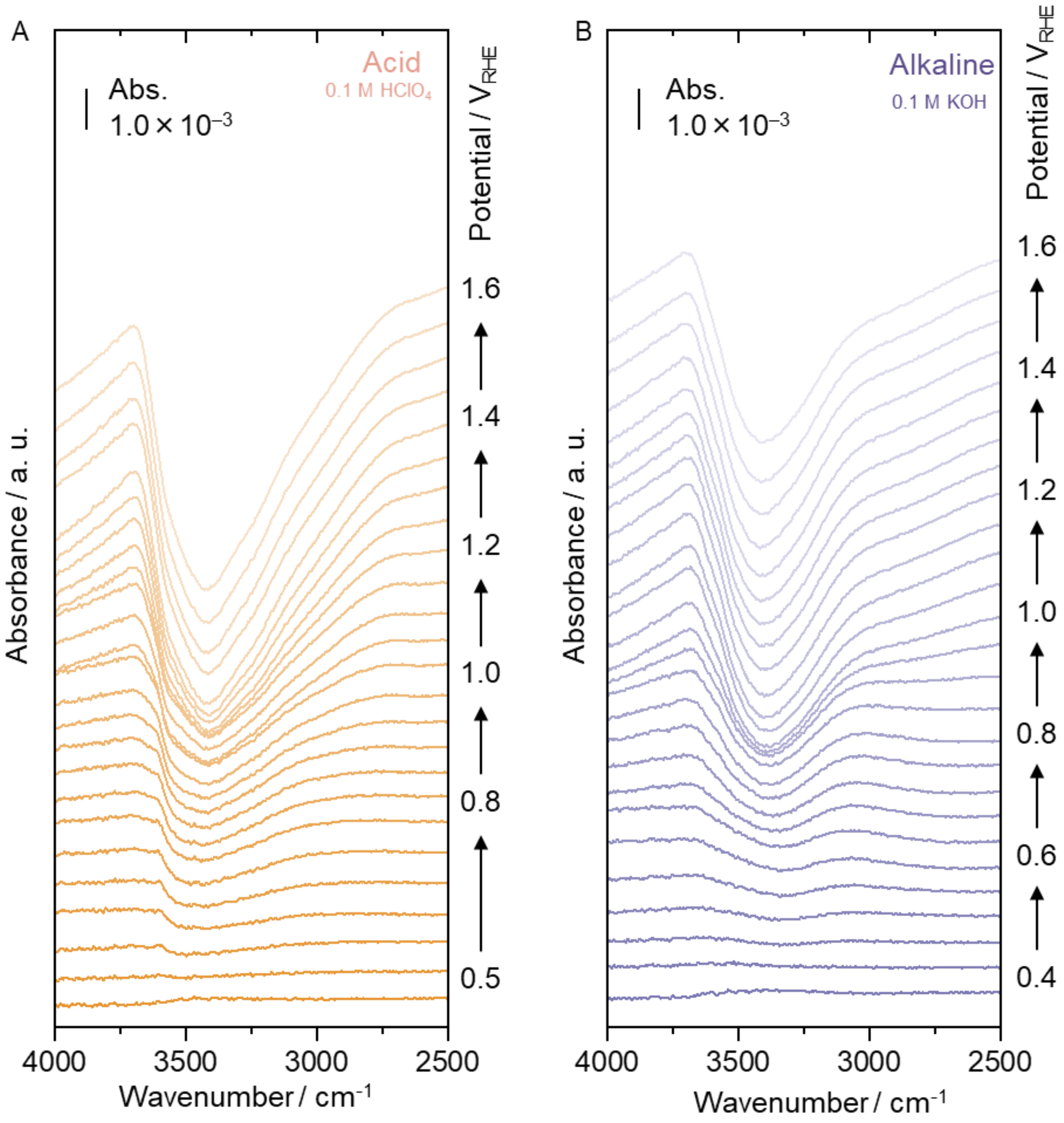

Figure S19 (A) ATR-SEIRAS spectra of the potential dependent behavior of interfacial water on IrO_x in 0.1 M $HClO_4$ solution at O-H stretching regime at every 50 mV. Reference spectra is taken at 0.5 V_{RHE}. Baseline of each spectra was corrected using OMNIC software with three-point auto correction method (B) ATR-SEIRAS spectra of the potential dependent behavior of interfacial water in 0.1 M KOH solution at every 50 mV. Reference spectra is taken at potential of 0.4 V_{RHE}.

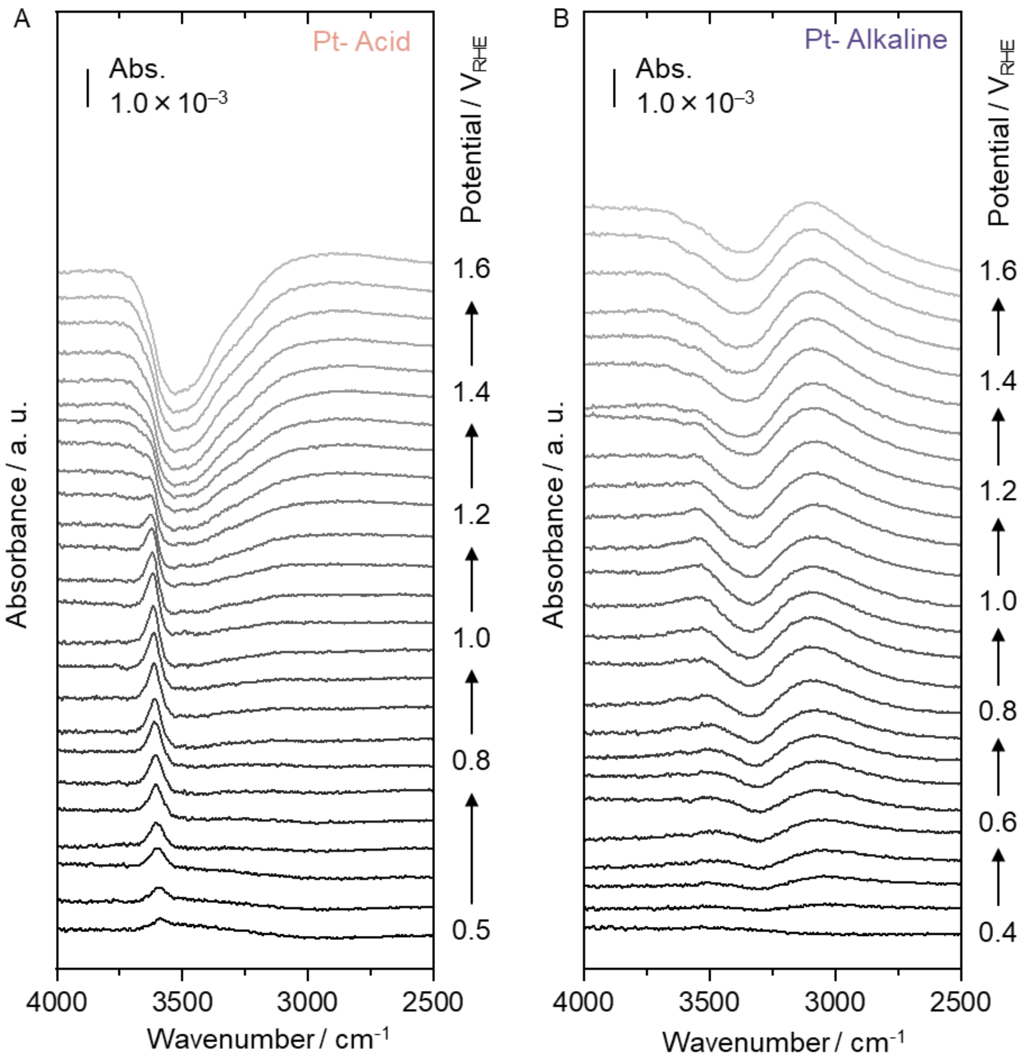

Figure S20 (A) ATR-SEIRAS spectra of Pt substrate in 0.1 M $HClO_4$ solution (A) and 0.1 M KOH solution (B) at every 50 mV. Reference spectra are taken at potential of 0.5 V and 0.4V_{RHE} for $HClO_4$ and KOH solution, respectively. Baseline of each spectrum was corrected using OMNIC software with three-point auto correction method.

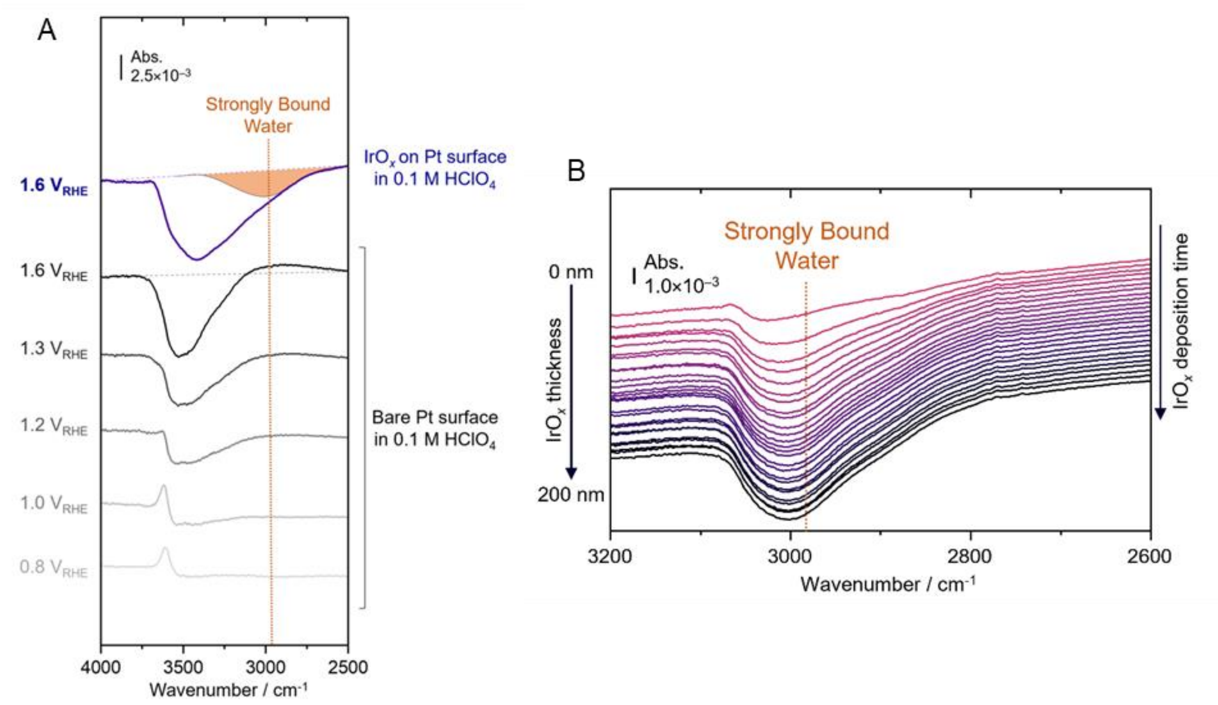

Figure S21 (A) ATR-SEIRAS spectra of the potential dependent behavior of interfacial water over Pt surface in 0.1 M $HClO_4$ solution at O-H stretching regime. Reference spectra is taken at 0.6 V_{RHE}. ATR-SEIRAS spectrum of IrO_x on Pt surface in 0.1 M $HClO_4$ solution at 1.6 V_{RHE} is also shown for comparison. The potential dependent spectra are very different in the absence of the IrO_x catalyst, suggesting that the changes observed are related to the catalyst surface. Furthermore, a strongly bound water feature is only visible with IrO_x. (B) ATR-SEIRAS spectra of the potential dependent behavior of interfacial water over Pt surface during IrO_x deposition. Reference spectra is taken at OCP in deposition solution.

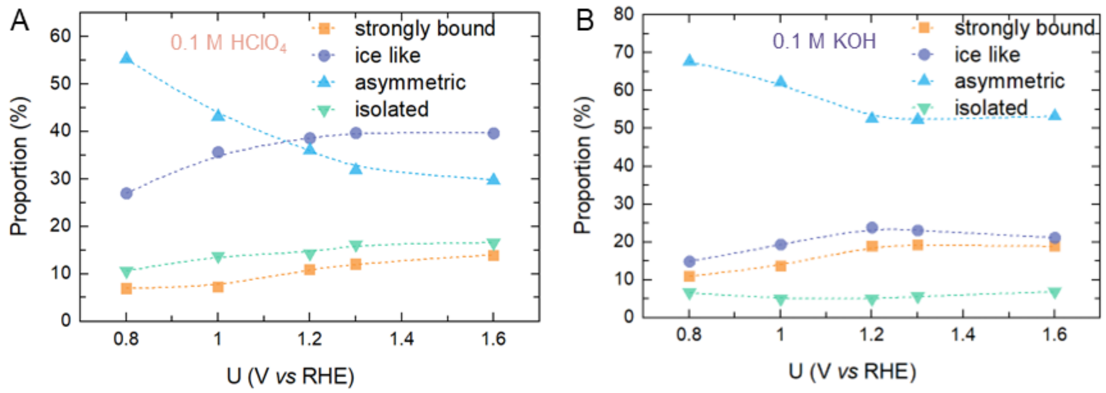

Figure S22 Quantification of the fraction of the different water species as a function of potential in acid (A) and alkaline (B). The fraction of each water species are determined by the area of its peaks after deconvolution. The deconvolution process of spectra at each potential following the same 4-Guassian fitting process, with the same boundary constrains and initial guess. The deconvolution scripts are attached in Data availability section.

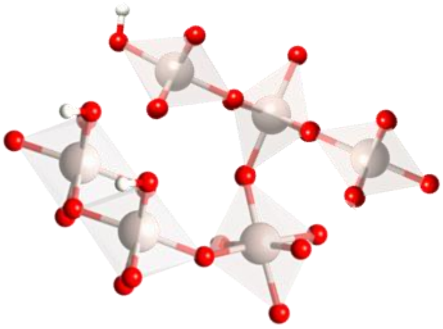

Figure S23 Possible short range ordered structure of amorphous IrO_x. Grey, red and white spheres represent iridium, oxygen and hydrogen atoms respectively. Compared with long range ordered rutile structure, amorphous IrO_x has randomly distributed corner-shared and edge-share IrO_6 units. The same proposed structure is reported in our previous work.[2]

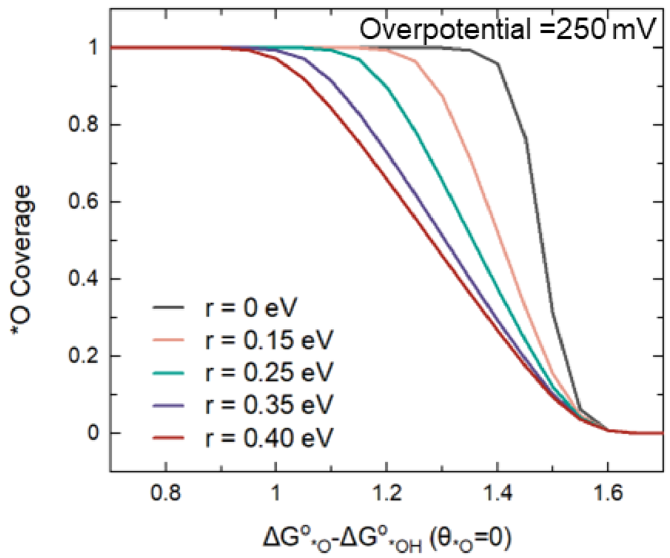

Figure S24 Coverage of *O for catalysts with different $\Delta G^{o}{}_{*O}$–$\Delta G^{o}{}_{*OH}(\theta_{*O}=0)$ and r values at a constant overpotential of 250 mV. The coverage is obtained by numerically solving the Frumkin isotherm equation at a given r and $\Delta G^{o}{}_{*O}$–$\Delta G^{o}{}_{*OH}(\theta_{*O}=0)$. The obtained coverage θ_{*O} is then use as input to determine the value of $\Delta G^{o}{}_{*O}$–$\Delta G^{o}{}_{*OH}(\theta_{*O})$ using Frumkin isotherm, i.e. $\Delta G^{0}{}_{(\theta)} = \Delta G^{0}{}_{(\theta=0)} + r \cdot \theta$. The coverage dependent $\Delta G^{o}{}_{*O}$–$\Delta G^{o}{}_{*OH}(\theta_{*O})$ is used to determine the thermodynamic overpotential of reaction, analogous to the construction of conventional volcano plot by DFT calculations.[12, 13]

(1) Bozal-Ginesta, C.; Rao, R. R.; Mesa, C. A.; Liu, X.; Hillman, S. A. J.; Stephens, I. E. L.; Durrant, J. R. Redox-State Kinetics in Water-Oxidation IrOx Electrocatalysts Measured by Operando Spectroelectrochemistry. *ACS Catalysis* **2021**, *11* (24), 15013-15025. DOI: 10.1021/acscatal.1c03290.
(2) Liang, C.; Rao, R.; Svane, K.; Hadden, J.; Moss, B.; Scott, S.; Sachs, M.; Murawski, J.; Frandsen, A.; Riley, J.; et al. Unravelling the effects of active site densities and energetics on the water oxidation activity of iridium oxides. **2023**. DOI: 10.21203/rs.3.rs-2605628/v1.
(3) Ravel, B.; Newville, M. ATHENA, ARTEMIS, HEPHAESTUS: data analysis for X-ray absorption spectroscopy using IFEFFIT. *Journal of synchrotron radiation* **2005**, *12* (4), 537-541.
(4) Diklić, N.; Clark, A. H.; Herranz, J.; Diercks, J. S.; Aegerter, D.; Nachtegaal, M.; Beard, A.; Schmidt, T. J. Potential Pitfalls in the Operando XAS Study of Oxygen Evolution Electrocatalysts. *ACS Energy Letters* **2022**, *7* (5), 1735-1740. DOI: 10.1021/acsenergylett.2c00727.
(5) Katayama, Y.; Nattino, F.; Giordano, L.; Hwang, J.; Rao, R. R.; Andreussi, O.; Marzari, N.; Shao-Horn, Y. An In Situ Surface-Enhanced Infrared Absorption Spectroscopy Study of Electrochemical CO2 Reduction: Selectivity Dependence on Surface C-Bound and O-Bound Reaction Intermediates. *The Journal of Physical Chemistry C* **2019**, *123* (10), 5951-5963. DOI: 10.1021/acs.jpcc.8b09598.
(6) Katayama, Y.; Okanishi, T.; Muroyama, H.; Matsui, T.; Eguchi, K. Enhanced Supply of Hydroxyl Species in CeO2-Modified Platinum Catalyst Studied by in Situ ATR-FTIR Spectroscopy. *ACS Catalysis* **2016**, *6* (3), 2026-2034. DOI: 10.1021/acscatal.6b00108.
(7) Katayama, Y.; Kubota, R.; Rao, R. R.; Hwang, J.; Giordano, L.; Morinaga, A.; Okanishi, T.; Muroyama, H.; Matsui, T.; Shao-Horn, Y.; et al. Direct Observation of Surface-Bound Intermediates During Methanol Oxidation on Platinum Under Alkaline Conditions. *The Journal of Physical Chemistry C* **2021**, *125* (48), 26321-26331. DOI: 10.1021/acs.jpcc.1c06878.
(8) Willinger, E.; Massué, C.; Schlögl, R.; Willinger, M. G. Identifying key structural features of IrOx water splitting catalysts. *Journal of the American Chemical Society* **2017**, *139* (34), 12093-12101.
(9) González-Flores, D.; Sánchez, I.; Zaharieva, I.; Klingan, K.; Heidkamp, J.; Chernev, P.; Menezes, P. W.; Driess, M.; Dau, H.; Montero, M. L. Heterogeneous Water Oxidation: Surface Activity versus

Amorphization Activation in Cobalt Phosphate Catalysts. *Angewandte Chemie International Edition* **2015**, *54* (8), 2472-2476. DOI: https://doi.org/10.1002/anie.201409333 (acccessed 2023/10/04).
(10) Klingan, K.; Ringleb, F.; Zaharieva, I.; Heidkamp, J.; Chernev, P.; Gonzalez-Flores, D.; Risch, M.; Fischer, A.; Dau, H. Water Oxidation by Amorphous Cobalt-Based Oxides: Volume Activity and Proton Transfer to Electrolyte Bases. *ChemSusChem* **2014**, *7* (5), 1301-1310. DOI: https://doi.org/10.1002/cssc.201301019 (acccessed 2023/10/04).
(11) Pasquini, C.; Zaharieva, I.; González-Flores, D.; Chernev, P.; Mohammadi, M. R.; Guidoni, L.; Smith, R. D. L.; Dau, H. H/D Isotope Effects Reveal Factors Controlling Catalytic Activity in Co-Based Oxides for Water Oxidation. *Journal of the American Chemical Society* **2019**, *141* (7), 2938-2948. DOI: 10.1021/jacs.8b10002.
(12) Rossmeisl, J.; Qu, Z. W.; Zhu, H.; Kroes, G. J.; Nørskov, J. K. Electrolysis of water on oxide surfaces. *Journal of Electroanalytical Chemistry* **2007**, *607* (1-2), 83-89. DOI: 10.1016/j.jelechem.2006.11.008.
(13) Man, I. C.; Su, H. Y.; Calle-Vallejo, F.; Hansen, H. A.; Martínez, J. I.; Inoglu, N. G.; Kitchin, J.; Jaramillo, T. F.; Nørskov, J. K.; Rossmeisl, J. Universality in Oxygen Evolution Electrocatalysis on Oxide Surfaces. *ChemCatChem* **2011**, *3* (7), 1159-1165. DOI: 10.1002/cctc.201000397.
